# Insights Into Drug Repurposing, as Well as Specificity and Compound Properties of Piperidine-Based SARS-CoV-2 PLpro Inhibitors

**DOI:** 10.3389/fchem.2022.861209

**Published:** 2022-04-12

**Authors:** Dale J. Calleja, Nathan Kuchel, Bernadine G. C. Lu, Richard W. Birkinshaw, Theresa Klemm, Marcel Doerflinger, James P. Cooney, Liana Mackiewicz, Amanda E. Au, Yu Q. Yap, Timothy R Blackmore, Kasiram Katneni, Elly Crighton, Janet Newman, Kate E. Jarman, Melissa J. Call, Bernhard C. Lechtenberg, Peter E. Czabotar, Marc Pellegrini, Susan A. Charman, Kym N. Lowes, Jeffrey P. Mitchell, Ueli Nachbur, Guillaume Lessene, David Komander

**Affiliations:** ^1^ Department of Medical Biology, Walter and Eliza Hall Institute, University of Melbourne, Melbourne, VIC, Australia; ^2^ Centre for Drug Candidate Optimisation, Monash Institute of Pharmaceutical Sciences, Monash University, Parkville, VIC, Australia; ^3^ Commonwealth Scientific and Industrial Research Organisation (CSIRO), Biomedical Program, Parkville, VIC, Australia; ^4^ Department of Pharmacology and Therapeutics, The University of Melbourne, Melbourne, VIC, Australia

**Keywords:** Nsp3, PLpro, inhibitor, SARS-CoV-2, repurposing, structure, ADME, COVID-19

## Abstract

The COVID-19 pandemic continues unabated, emphasizing the need for additional antiviral treatment options to prevent hospitalization and death of patients infected with SARS-CoV-2. The papain-like protease (PLpro) domain is part of the SARS-CoV-2 non-structural protein (nsp)-3, and represents an essential protease and validated drug target for preventing viral replication. PLpro moonlights as a deubiquitinating (DUB) and deISGylating enzyme, enabling adaptation of a DUB high throughput (HTS) screen to identify PLpro inhibitors. Drug repurposing has been a major focus through the COVID-19 pandemic as it may provide a fast and efficient route for identifying clinic-ready, safe-in-human antivirals. We here report our effort to identify PLpro inhibitors by screening the ReFRAME library of 11,804 compounds, showing that none inhibit PLpro with any reasonable activity or specificity to justify further progression towards the clinic. We also report our latest efforts to improve piperidine-scaffold inhibitors, *5c* and *3k*, originally developed for SARS-CoV PLpro. We report molecular details of binding and selectivity, as well as *in vitro* absorption, distribution, metabolism and excretion (ADME) studies of this scaffold. A co-crystal structure of SARS-CoV-2 PLpro bound to inhibitor *3k* guides medicinal chemistry efforts to improve binding and ADME characteristics. We arrive at compounds with improved and favorable solubility and stability characteristics that are tested for inhibiting viral replication. Whilst still requiring significant improvement, our optimized small molecule inhibitors of PLpro display decent antiviral activity in an *in vitro* SARS-CoV-2 infection model, justifying further optimization.

## Introduction

The COVID-19 pandemic continues unabated in many countries, and while large-scale vaccination efforts are underway, the management of population health, economic impact and as-of-yet unknown long-term effects on physical and mental health will be a key challenge for the next decade. To truly overcome the threat posed by the causative coronavirus (CoV), SARS-CoV-2, and its emerging variants of concern, it is paramount to generate and clinically validate additional, orthogonally acting antiviral drugs (Dolgin, 2021). We envisage that small molecule drugs that target the viral proteins themselves, acting in concert with vaccination, will stop viral replication in cells and hence impact on virus fitness and transmission (Dolgin, 2021). Such drugs will act to treat established disease but, perhaps more importantly, also work as a prophylaxis to prevent disease in high-risk populations. The targets required for such small molecule drugs are well established: the CoV genome comprises non-structural proteins (nsps) that each fulfills (an) essential function(s), and therefore offer a host of putative targets (Hartenian et al., 2020). Several stand out based upon essentiality, druggability and proof-of-concept work performed (Gao et al., 2020; Hillen et al., 2020; Hoffmann et al., 2020; Klemm et al., 2020; Shin et al., 2020; Zhang et al., 2020; Zhou et al., 2020). These include the viral replicase, comprised of several nsps that recombine after production to assemble the viral machinery responsible for carbon-copying viral genetic material (Subissi et al., 2014; Malone et al., 2022), as well as two essential proteases, nsp3/PLpro and nsp5/Mpro responsible for releasing individual nsps from the viral polyprotein (Hartenian et al., 2020). Whereas nsp3/PLpro is responsible for releasing the first four nsps, Mpro generates nsp5 to nsp16 (Fan et al., 2004; Harcourt et al., 2004).

PLpro refers to the protease domain within the 1945 amino acid (aa) multi-domain protein nsp3. As a conserved papain-like Cys protease from the C16 family (Rawlings et al., 2012), PLpro hydrolyses amino acid sequences with a specific Leu-Xaa-Gly-Gly motif, found at the junctions between nsp1/2, nsp2/3 and nsp3/4 (where Xaa is Asn, Lys or Lys, respectively) (Rut et al., 2020). Importantly, the same motif is present within a subset of human proteins that are also targeted by PLpro/nsp3; most notable are the C-terminus of human ubiquitin and the ubiquitin-like modifier, Interferon Stimulated Gene 15 (ISG15) that comprise a Leu-Arg-Gly-Gly motif. Indeed, ubiquitin and ISG15 are intricately involved in the human anti-viral response (Heaton et al., 2016; Perng and Lenschow, 2018), enabling the virus to directly interfere with host signalling processes. Moreover, the fact that PLpro also acts as a deubiquitinase (DUB) and deISGylase, enables the exploitation of many tools and assays to measure PLpro activity (Hassiepen et al., 2007; Hospenthal et al., 2015; Gui et al., 2020).

The PLpro enzyme of previous CoVs, in particular SARS-CoV and MERS-CoV have been studied in great detail by the teams of Andrew Mesecar, Scott Pegan, Chris Lima and others (Harcourt et al., 2004; Barretto et al., 2005; Lindner et al., 2005; Ratia et al., 2006; Ratia et al., 2008; Ghosh et al., 2009; Ghosh et al., 2010; Báez-Santos et al., 2014; Lee et al., 2015; Békés et al., 2016). We and others identified many of the previously described features also in SARS-CoV-2 PLpro, including its essentiality in viral replication (Freitas et al., 2020; Klemm et al., 2020; Rut et al., 2020; Shin et al., 2020). Indeed, SARS-CoV and SARS-CoV-2 PLpro share 82% sequence identity (Freitas et al., 2020; Klemm et al., 2020; Rut et al., 2020; Shin et al., 2020).

Inhibitor development campaigns against SARS-CoV PLpro have resulted in two main chemical scaffolds (Ratia et al., 2008; Ghosh et al., 2009; Ghosh et al., 2010; Báez-Santos et al., 2014) the benzamide ring (“***GRL-0617***” family of compounds) and the piperidine carboxamide (“***5c***” family of compounds) series. Both have undergone considerable medicinal chemistry efforts to arrive at compounds with sub-micromolar *in vitro* inhibitory activity (Ghosh et al., 2009; Ghosh et al., 2010; Báez-Santos et al., 2014). As the SARS-CoV and MERS-CoV epidemics subsided, unfortunately so did the development of inhibitors identified in early drug discovery campaigns.

The SARS-CoV-2 pandemic reignited PLpro drug discovery in two areas. Many efforts focused on drug repurposing, aiming to identify a PLpro inhibitor within the already approved drugs and drug candidates available. The benefits of this approach are often mistakenly considered as to provide an immediate starting point for clinical studies, and it is important to recognise that it does not alleviate the need for pre-clinical development (Pushpakom et al., 2019; Begley et al., 2021). The urgency of the COVID-19 pandemic nonetheless justified this avenue of exploration on the exceedingly small chance that potent drugs optimized for one target may be equally potent against new targets. We critically discuss the reported results from putative PLpro inhibitors identified from repurposing approaches in our associated Review (Calleja et al., this issue).

Secondly, we and others showed that ***GRL-0617*** and ***5c*** compounds could efficiently block SARS-CoV-2 PLpro both *in vitro* and in cells and stop viral replication in cell culture (Klemm et al., 2020; Shin et al., 2020). Efficacy of these early lead compounds was promising but required improvements. Several papers have by now described iterations of the ***GRL-0617*** series compounds (Ma et al., 2021; Osipiuk et al., 2021; Shen et al., 2021), for details, see our associated Review on these drug discovery efforts (Calleja et al., this issue).

Here, we present our efforts to identify PLpro inhibitors within the ReFRAME compound library (Janes et al., 2018), showing that none are suitable for further development. Secondly, we update on our efforts to characterize the ***5c*** scaffold of PLpro inhibitors we first described in Klemm et al. (2020). A co-crystal structure of PLpro bound to the related compound, ***3k***, and additional analyses explain compound specificity, however, compound stability profiling on ***5c*** identified numerous metabolic liabilities. A medicinal chemistry campaign with the aim to improve compound properties (efficacy and stability) resulted in compounds with the same potency as ***5c*,** but with improved ADME properties. These preliminary studies indicate that significant improvements are still required to arrive at a lead candidate.

## Results

### Testing ReFRAME Compounds Against PLpro

Most published activity-based PLpro assays measure cleavage of a FRET-labelled peptide substrate based on a native cleavage sequence such as the C-terminus of ubiquitin or ISG15 (LRLRGG). An alternative assay for PLpro assesses hydrolysis of ubiquitin-Rhodamine110; Rhodamine110 is cleaved not off a peptide but off the 8.5 kDa ubiquitin moiety. PLpro binds to ubiquitin-Rhodamine110 more tightly when compared to peptides as it interacts with a significant portion of the 8,000 Å^2^ ubiquitin surface (see our associated Review for a discussion on assay design). In our previous work (Klemm et al., 2020), we adapted a ubiquitin-Rhodamine110-based high throughput screening (HTS) assay to identify small molecule PLpro inhibitors as previously developed for human DUBs (Turnbull et al., 2017). A first drug repurposing campaign was performed, in the hope to uncover human-safe medications that could be progressed towards the clinic. We ideally required nanomolar inhibitory activity, a “clean” specificity profile against human DUBs (Turnbull et al., 2017) and sensible chemistry lacking reactive groups or PAINS (Baell and Holloway, 2010). However, screening 5,576 molecules including 3,727 unique FDA approved small molecule drugs, we failed to identify suitable compounds that would enable progression to the clinic (Klemm et al., 2020).

**FIGURE 1:**
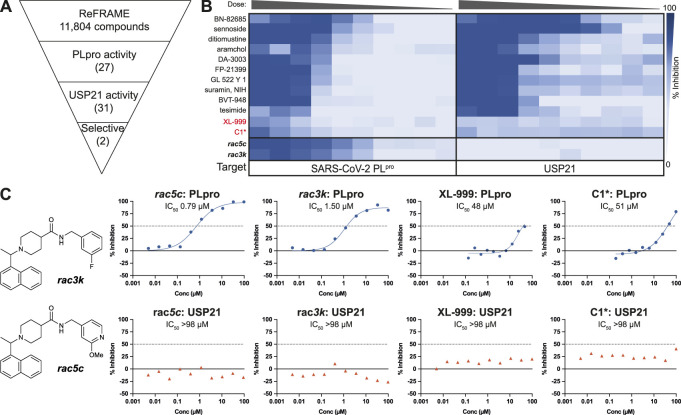
High Throughput Screen (HTS) of the ReFRAME library for inhibitors of SARS-CoV-2 PLpro. **(A)** Schematic showing the screening cascade for the identification of selective PLpro inhibitors. 11,804 compounds were screened at 8.33, 4.17 or 0.83 µM through a primary screen against PLpro. Hits showing a median absolute deviation >2.5 (over the DMSO negative control) were selected for a 10-point titration assays against PLpro and a counter screen against USP21 (5 nM). Of the 53 total hits from the screen, 27 compounds retested showing activity against PLpro in the 10-point titration, and 31 showed activity against USP21. Two compounds, XL-999 and C1*, were found to be selective for PLpro over USP21. **(B)** 10-point titration for the top 12 compounds showing activity against SARS-CoV-2 PLpro. Compounds were assayed at a top concentration of 100 µM and titrated using 1:2 (PLpro) or 1:3 (USP21) serial dilutions. 100 µM ***rac5c*** was used as a positive control for the HTS. 10-point titration curves of ***rac5c*** and ***rac3k*** were performed in a separate control assay and are shown for comparison. **(C)** Two selective hits from the screening of the ReFRAME library were XL-999 and C1*, with an IC_50_ of 48 and 51 μM, respectively. Both were found to be weak inhibitors of PLpro and were not further investigated. 10-point titration curves of ***rac5c*** and ***rac3k*** were performed in a separate control assay and are shown for comparison. IC_50_ values were derived from one set of independent experiments (*n* = 1).

We now extended these studies to include the ReFRAME library (Janes et al., 2018), which is a collection of 11,804 compounds, mostly approved drugs and drug candidates that had progressed to late-stage clinical trials, and hence had in-human safety data associated (Janes et al., 2018). As before, our PLpro HTS yielded excellent and highly robust, reproducible data (Figure 1, Supplementary Figure S1). 53 compounds passed the primary screen, and 27 showed inhibitory potential in 10-point titration studies (Figures 1A,B, Supplementary Figure S1D). The latter were also tested against USP21 as a selectivity counter screen. All but two compounds showed identical inhibition towards USP21, indicating off-target issues, compound reactivity, and/or assay interference (Figure 1B, Supplementary Figure S1D). The two remaining compounds were XL-999, a receptor tyrosine kinase and FLT3 kinase inhibitor (DrugBank (Wishart et al., 2008) ID: DB05014), and a derivative of codeine, an opioid receptor agonist (Figure 1C). Both compounds displayed only weak *in vitro* inhibitory activity against PLpro (IC_50_ 48 and 51 μM, respectively) (Figure 1C) and had been optimized for their human targets. Weak activity against PLpro (necessitating extreme dosing regimes) rendered both compounds unsuitable for progression towards the clinic. Compounds were also considered unsuitable as starting points for medicinal chemistry due to inferior potency and ligand efficiency when compared to other scaffolds (see below).

While we performed these studies, a second group also reported screening of SARS-CoV-2 PLpro against the ReFRAME library (Redhead et al., 2021). The best compound in their assays, Tarloxotinib, demonstrated inhibitory activity against PLpro, and strikingly, in a separate set of experiments also inhibited Mpro. In our PLpro assay, Tarloxotinib did not show any inhibitory activity (Supplementary Figure S1). Both proteases hold very different active sites such that appropriate orthogonal assays must be performed when identifying potential Mpro/PLpro dual-inhibitors. In the mentioned study, Tarloxotinib identification as a hit was not followed up with any counter screens against other human DUBs, nor direct binding assays against PLpro.

Together, based on our own results and published studies (Klemm et al., 2020; Redhead et al., 2021), we conclude that drug repurposing against PLpro is not feasible. Moreover, the premise to arrive at immediate treatments appears somewhat flawed since any compound repurposing would still require extensive pre-clinical development for a new indication. Our assessment (further elaborated in our associated Review in this Issue of Frontiers in Chemistry) is in line with work in other therapeutic areas, as highlighted recently (Begley et al., 2021).

#### Further Characterization of Piperidine Scaffold PLpro Inhibitors, 3k and 5c

Drug repurposing by us and others failed to uncover compounds that could progress to the clinic, and while some of the structurally characterized hits reported by others may serve as potential starting points, we chose to focus on and further characterize the more amenable sub-µM piperidine based inhibitors previously reported for SARS-CoV and SARS-CoV-2 PLpro, namely the ***5c*** family of compounds. In our earlier work (Klemm et al., 2020), we described the effects of compounds ***rac5c*** and ***rac3k*** (Figure 1C **,** Supplementary Figure S2), which inhibited PLpro with an IC_50_ of 600–800 nM, and which decreased SARS-CoV-2 viral titers (TCID50) by 2-3 orders of magnitude when tested at 11 µM concentration in a cellular infection model, which is comparable to Remdesivir at 12.5 µM (Klemm et al., 2020).

Reported compounds ***5c*** and ***3k*** contain a stereocenter between the naphthalene and piperidine rings. In our earlier study, we used racemic mixtures, ***rac5c*** and ***rac3k***. Previous work on SARS-CoV PLpro described the (R)-enantiomer as having improved activity when compared to the (S)-enantiomer (Báez-Santos et al., 2014) We synthesized and tested the (R)-enantiomers of both ***5c*** and ***3k*** *in vitro* and the results showed no loss in inhibitory activity over their racemic counterparts (Supplementary Figure S2A)**.** For the remainder of the study, we used the (R)-enantiomers of the compound series and refer to them as ***3k*** and ***5c***. As most studies for SARS-CoV-2 PLpro focused on optimizing the inhibitor ***GRL-0617*** (discussed in our associated Review) we compared PLpro inhibition by ***GRL-0617*** to the ***5c*** family of compounds. We observed similar IC_50_ values (Supplementary Figure S2B) to those reported in other studies (Fu et al., 2021; Ma et al., 2021; Osipiuk et al., 2021; Shen et al., 2021) and confirmed observations from the original SARS-CoV work that ***5c*** remains a more potent inhibitor of SARS-CoV-2 PLpro when compared to ***GRL-0617*** (Supplementary Figure S2)**.**

**FIGURE 2:**
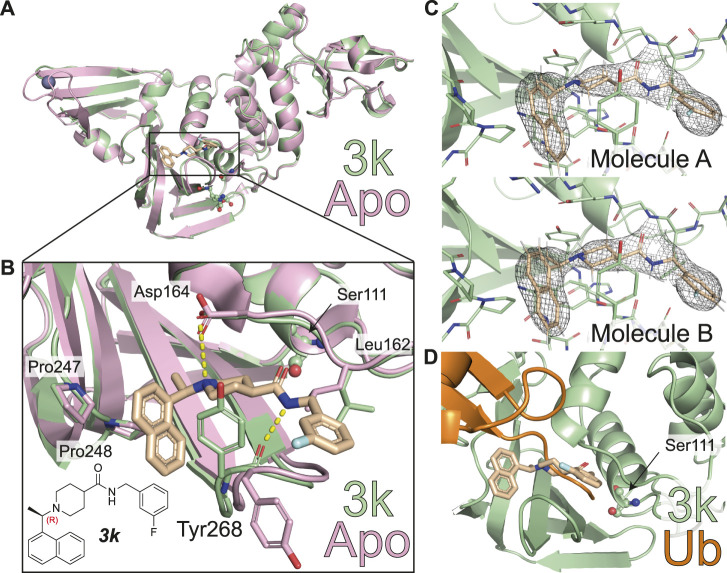
Molecular basis for inhibition of SARS-CoV-2 PLpro by ***3k***. **(A)** Structure of SARS-CoV-2 PLpro bound to ***3k*** in green, with inhibitor in wheat colour in ball-and stick representation representing the (R)-enantiomer. A superimposed structure of apo PLpro [pink, PDB 6WZU (Osipiuk et al., 2021)] shows that the inhibitor does not induce global conformational changes. Catalytic residues are shown in ball and stick representation, and a bound zinc ion in apo PLpro is shown as a grey sphere. **(B)** Close-up view of the ligand binding site for ***3k*** with key residues indicated. The chemical structure of ***3k*** is also shown, with the stereocenter labelled in red. Hydrogen bonds are indicated by yellow dotted lines. **(C)** 2|Fo| – |Fc| electron density map contoured at 1 σ for ***3k*** of molecule A (top) and molecule B (bottom) in the asymmetric unit. Also see Supplementary Figure S3. **(D)** Close-up view of the ligand binding site for inhibitor ***3k*** overlaid with ubiquitin-bound PLpro in orange [PLpro ∼ Ub, PDB 6XAA (Klemm et al., 2020)]. The catalytic Cys111 of PLpro was mutated to a Ser (C111S) in the compound complex. ***3k*** binding inhibits PLpro catalytic activity by blocking the C-terminus of Ub or ISG15 entering the catalytic cleft.

**FIGURE 3:**
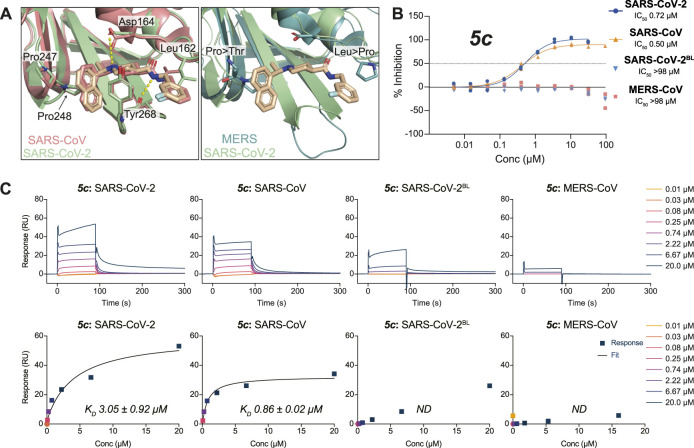
Molecular basis for the inhibitor specificity towards PLpro variants. **(A)** Close-up view of the overlay between the ligand binding sites of ***3k*** in complex with SARS-CoV-2 PLpro (green) and SARS-CoV PLpro in salmon (PDB 4OW0 (Báez-Santos et al., 2014)) or MERS-CoV PLpro in teal (PDB 4RNA (Lee et al., 2015)). Cross reactivity by ***3k*** between species is a consequence from the conservation of key interacting residues as indicated. Crucial differences in interacting residues underpin compound specificity. **(B)** ***5c*** was tested for specificity to inhibit SARS-CoV PLpro, MERS-CoV PLpro, SARS-CoV-2 PLpro, or SARS-CoV-2 PLpro with residues 267–272 of blocking loop 2 (BL2) substituted for those in MERS-CoV (SARS-CoV-2^BL^) in a UbRh assay. Inhibitor ***5c*** is cross-reactive with SARS-CoV PLpro and SARS-CoV-2 PLpro but not with MERS-CoV PLpro. These data indicate that engaging the conserved BL2 is crucial for the inhibition of PLpro by ***5c***. Experiments were performed using the HTS assay as two independent experiments (*n* = 2) each containing two technical replicates. Individual data points represent the mean replicate value for each experiment. **(C)** SPR assays for compound ***5c*** against PLpro variants from **(B)**. The top panels show double referenced sensorgram data as a function of time and the bottom show steady-state dose response curves. Absence of inhibitor ***5c*** cross-reactivity with MERS-CoV PLpro can be explained by a loss in direct binding. Minor binding can be detected when assayed against SARS-CoV-2^BL^ PLpro compared with MERS-CoV PLpro and confirms most free energy loss during binding is resulting from interactions with the conserved BL2. SPR data for ***3k*** can be found in Supplementary Figure S5. All SPR experiments were performed in triplicate; a representative example is shown. See Supplementary Table S1 for all data and Supplementary Figure S9 for the response curves of the remaining experiments.

The structure of ***3k*** bound to SARS-CoV PLpro is published (PDB 4OW0) (Báez-Santos et al., 2014). We co-crystallized ***3k*** with a mutant form of SARS-CoV-2 PLpro in which the catalytic Cys111 was changed to Ser (PLpro^C111S^), which we and others found to yield a more stable enzyme (Osipiuk et al., 2021). The best crystal diffracted to 2.66 Å, and structure determination by molecular replacement revealed the structure of the PLpro bound to ***3k*** (Table 1; Figures 2, 3, Supplementary Figure S3). The new crystal form (space group *P*2_1_2_1_2, see a list of all SARS-CoV-2 crystal forms in our associated Review) has two molecules in the asymmetric unit; both molecules are superimposable with an RMSD of 0.51 Å, and show excellent electron density for the ligand in identical ligand binding sites (Figure 2C **,** Supplementary Figures S3B,C).

**TABLE 1:** Data collection and refinement statistics. Values in parentheses are for highest-resolution shell.

	SARS-CoV-2 PLpro bound to inhibitor *3k*
**Data collection**	
Space group	*P* 2_1_ 2_1_ 2
Cell dimensions	
*a*, *b*, *c* (Å)	72.951, 90.632, 99.766
*α, β, γ* (°)	90.00, 90.00, 90.00
Resolution (Å)	38.49 – 2.66 (2.76 – 2.66)
*R*_merge_ (within I+/I-)	0.062 (0.608)
*< I/σI >*	7.0 (0.9)
Completeness (%)	99.8 (98.9)
Redundancy	2.0 (2.0)
**Refinement**	
Resolution (Å)	38.49 – 2.66
No. reflections	19521
*R*_work_/*R*_free_	0.200/0.257
No. atoms	
Protein	4792
Ligand/ion	141
Water	31
*B*-factors	
Protein	57.4
Ligand/ion	69.8
Water	46.0
R.m.s deviations	
Bond lengths (Å)	0.005
Bond angles (°)	0.81

The new crystal structure contributes to the understanding of how ***3k*** and ***5c*** inhibit SARS-CoV-2 PLpro and confirm many of the aspects previously illuminated in the SARS-CoV PLpro complex structures of the same series (Báez-Santos et al., 2014). Firstly, compound binding does not invoke gross conformational perturbation of the PLpro fold when compared to apo or ubiquitin-bound PLpro (RMSD 0.44 Å compared to apo PLpro, PDB 6WRH (Osipiuk et al., 2021) and 0.48 Å compared to ubiquitin-bound PLpro PDB 6XAA (Klemm et al., 2020) (Figure 2A). The (R)-enantiomer of the compound was used for co-crystallisation and lies in the binding site (Figure 2B). ***3k*** occupies the channel required by the enzyme to hold to the C-terminal tail of ubiquitin and ISG15, which is on two sides lined by the static core of the PLpro Thumb domain and held in place by the more flexible blocking loop (aa 267-272), termed BL2, an extended β-hairpin that folds over the compound binding site (Figures 2B,D). Tyr268 at the turn of BL2 restrains the central piperidine ring, almost entirely burying it in the enzyme; the piperidine amino group further forms a hydrogen bond with the side chain of Asp164 of the Thumb domain. The naphthyl ring extends into a hydrophobic pocket towards the ubiquitin binding bowl in PLpro, packing against Pro247 and Pro248 (Figure 2B). On the other side of the molecule extending towards the catalytic Cys, a substituted phenyl group is connected to the *para*-position of the piperidine ring by an amide-linker that forms interactions with both the domain and BL2, including through a key hydrogen bond between the compound amide and the backbone carbonyl of BL2 Tyr268. The substituted phenyl ring extends outwards from the domain core, due to side-chain rotation of Leu162, which blocks the path to the catalytic Cys111 (Figures 2B,D). As a result, ***3k*** and related compounds appear to wrap around BL2, remotely from the catalytic Cys111 (closest compound distance 9.7 Å), yet directly competing with ubiquitin/Ubl-tail binding to the protease channel. There are no sequence differences in residues lining the compound binding site between SARS-CoV and SARS-CoV-2 PLpro, and all interactions described here for SARS-CoV-2 PLpro with ***3k*** were seen in the previous structures of SARS-CoV PLpro with ***3k*** (Báez-Santos et al., 2014) (Figure 3A).

#### Molecular Basis for Compound Specificity Towards SARS-CoV and SARS-CoV-2 PLpro

To better understand cross-CoV PLpro specificity, and ideally identify or engineer a cross-specific inhibitor, we expanded our assay platform and routinely included SARS-CoV and MERS-CoV PLpro. The BL2 sequence is a poorly conserved region of CoV PLpro, and explains the inability of MERS-CoV PLpro to bind to and be inhibited by SARS-CoV PLpro inhibitors including ***5c*** and ***3k*** (Lee et al., 2015) (Figures 3A,B). To investigate these observations in the context of SARS-CoV-2 PLpro we also engineered a SARS-CoV-2 variant in which the BL2 loop was changed to the equivalent sequence of MERS-CoV. In this variant termed SARS-CoV-2 PLpro^BL^, the 4-amino acid (aa) BL2 loop of SARS-CoV-2 PLpro (G-NYQC-G) was replaced with the 5-aa sequence of MERS-CoV PLpro (G-IETAV-G) that lacks Tyr268. SARS-CoV-2 PLpro^BL^ shows lower activity compared to wild-type PLpro in ISG15 and tri-ubiquitin-cleavage assays (Supplementary Figure S4), yet all enzymes performed similarly in the ubiquitin-Rhodamine110 assay used in our HTS platform (data not shown). As anticipated, ***5c*** and ***3k*** inhibited SARS-CoV and SARS-CoV-2 PLpro similarly but failed to act on MERS-CoV PLpro or the SARS-CoV-2 PLpro^BL^ variant (Figure 3C, Supplementary Figure S5A, Table 2).

**TABLE 2:** Inhibitory activity based on HTS screen (IC_50_) and binding constants based on SPR (K_D_) for ***3k*, *5c*** and ***9***. SPR data for ***3k*** and ***9*** can be found in Supplementary Figures S5, S8, Supplementary Table S1.

	*3k*		*5c*		*9*	
	IC_50_ (µM)	K_D_ (µM)	IC_50_ (µM)	K_D_ (µM)	IC_50_ (µM)	K_D_ (µM)
SARS-CoV-2	1.02	2.40 ± 0.43	0.72	3.05 ± 0.92	0.76	1.86 ± 0.46
SARS-CoV	0.86	0.71 ± 0.07	0.50	0.86 ± 0.02	0.70	0.82 ± 0.02
MERS-CoV	>98	ND	>98	ND	>98	ND
SARS-CoV-2^BL^	>98	ND	>98	ND	>98	ND

NT, not tested; ND, not detected.

To confirm direct binding and learn about binding characteristics of our inhibitory compounds to PLpro, we established a surface plasmon resonance (SPR) platform using PLpro of SARS-CoV, SARS-CoV-2 and MERS-CoV, as well as the SARS-CoV-2 PLpro^BL^ mutant in parallel. For ***5c*** and ***3k***, results confirmed those obtained in the biochemical screening assays, also confirming that ***5c*** and ***3k*** lost binding to SARS-CoV-2 PLpro^BL^ (Figure 3C, Supplementary Figure S5B, Table 2).

**FIGURE 4:**
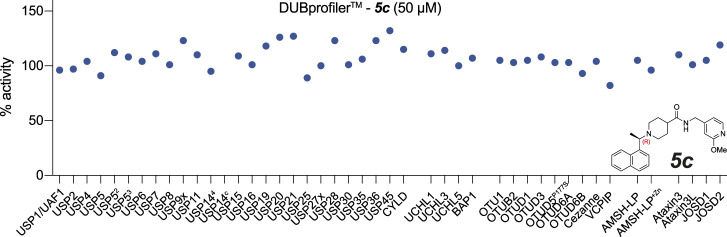
Inhibitor ***5c*** does not cross react with human DUBs. ***5c*** was screened at 50 µM against a commercial DUB specificity panel (Ubiquigent) that included several human USP family enzymes that are structurally the most similar DUB enzymes to PLpro. ***5c*** does not notably inhibit any of the tested human DUBs at 50 µM concentration. USP5^2^, USP5 assay performed with addition of ubiquitin at K_D_; USP5^3^, USP5 assay performed with addition of ubiquitin at B_max_. USP14^4^ indicates assay performed in the presence of proteasome-vinyl sulfone at K_D_. USP14^c^ indicates the proteasome-vinyl sulfone control only without USP14. See Methods.

Finally, we assessed the activity of the compounds against a panel of human DUBs, since specificity for PLpro over related human DUBs is essential to avoid toxicity issues. While PLpro is dissimilar at the sequence and structural level to any human DUB family, all known DUBs bind ubiquitin *via* the extended C-terminus. The selectivity of ***3k*** had been studied to a limited extent against a small panel of representative DUBs (Báez-Santos et al., 2014). We extended these studies by testing ***5c*** with a commercial DUB panel comprising 41 enzymes from all DUB families, assessed with a ubiquitin-Rhodamine110 assay (Figure 4). The results showed that ***5c***, at 50 μM, did not inhibit any of the human DUBs tested (Figure 4). The clean specificity profile of ***5c*** alleviates some concerns regarding off-target toxicity in human cells and tissues.

#### Elaboration and Improvement of Piperidine-Based PLpro Inhibitors

To improve compound properties, a medicinal chemistry campaign was initiated, focusing on key aspects of the compound. We learned from previous published works that attempted to improve the piperidine scaffold for SARS-CoV PLpro (Báez-Santos et al., 2014; Báez-Santos et al., 2015; Ghosh et al., 2020), enabling us to explore novel chemical space. We also knew that ***3k*** and ***5c*** were metabolically labile (discussed below) and our designs also aimed to improve the ADME properties of compounds.

**FIGURE 5:**
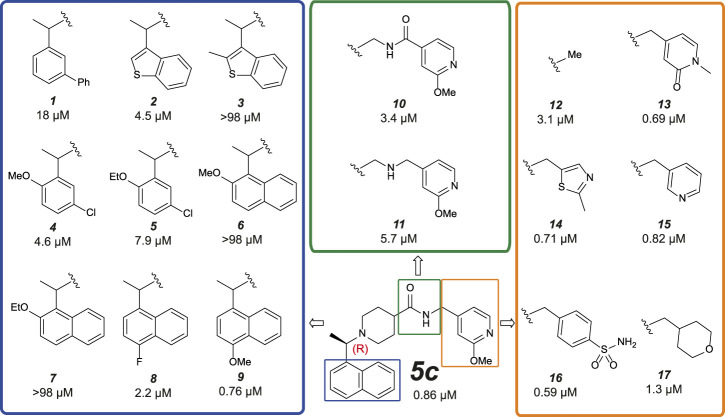
Medicinal Chemistry elaborations to improve piperidine based PLpro inhibitors. Subset of tested piperidine based PLpro inhibitors molecules indicating how the ***5c*** scaffold was altered. The blue box shows alterations to the naphthyl ring, the green box shows modifications of the amide linkage and the orange box shows a subset of the most potent alterations to the benzylic group. These data exposed key insights for improving inhibitor ***5c*** (i) tight SAR is evident around the naphthyl position, with only minor modifications able to achieve <1 µM activity; (ii) the amide and its positioning is important to compound activity; (iii) modifications at the benzylic group gave the most potent compounds. This position appears the most malleable to achieve improved potency against SARS-CoV-2 PLpro. The calculated IC_50_ (µM) is noted below each compound and is the average of two or four (***9*** only) independent experiments (n = 2) each containing two technical replicates. Dose response curves can be found in Supplementary Figures S6, S8 **.**

In our attempts to improve both potency against SARS-CoV-2 PLpro and metabolic stability, we generated more than 250 derivatives of the ***5c*** series of compounds (a selection of which is shown in Figure 5). All generated compounds were tested in our HTS platform against SARS-CoV-2 PLpro and counter screened against USP21 (Supplementary Figure S6). Selected compounds were also screened against other PLpro enzymes and underwent SPR characterization.

Naphthyl rings are often considered an undesirable functional group as they hold numerous metabolic liabilities including increased lipophilicity, and are considered possible toxicophores. Thus, we hypothesized the naphthyl ring was a significant contributor to the overall metabolic liability of these compounds and hence the primary target for optimization. Given that the π-stack arrangement with Pro247, Pro248 and Tyr268 of the blocking loop is known to be a significant contributor in ***5c*** binding to the SARS-CoV PLpro (Ratia et al., 2008; Báez-Santos et al., 2014) we sought to replicate this interaction with isosteric replacements. Based on the observation that ***5c*** and ***rac5c*** are equipotent in the primary assay (Supplementary Figure S2A) (Klemm et al., 2020) and for ease of synthesis, modifications to the naphthyl were initially prepared and tested as racemates (blue in Figure 5). Previously reported isosteric quinoline modifications (Báez-Santos et al., 2014) which maintained modest activity towards SARS-CoV PLpro were not comparable to the potency afforded by the naphthyl ring and were thus avoided. Replacement with a biphenyl (***1***, for compound numbering, refer to Figure 5), decreased potency 20-fold. Likewise, isosteric replacement with a benzothiophene moiety in ***2*** negatively impacted the potency whilst the substituted benzothiophene ***3*** was completely inactive. Surprisingly, simpler di-subsituted phenyl rings (compounds ***4*** and ***5***) maintained modest activity. However, the similar methoxy and ethoxy ortho substitutions on the naphtyl ring were not tolerated (e.g., ***6*** and ***7***). The fluorine substitution in ***8*** resulted in 3-fold activity loss while the methoxy derivative ***9*** showed comparative activity to the parent ***5c***; together, this data suggests that the interaction pocket for the naphthyl doesn’t tolerate electron deficient substituents but may tolerate electron-donating ones.

Next, we turned our attention to the amide bond (green box in Figure 5). Structural information suggests the amide carbonyl forms no key interaction with the protein. However, reversal of the amide bond ***10*** proved 4-fold less potent than the parent ***5c***. The amino analogue ***11*** led to an 7-fold drop in potency. Finally, we looked at optimizing the terminal benzyl group (orange box in Figure 5). Deletion analogue ***12*** resulted in only a 4-fold loss of potency suggesting that the existing substituents at this position contribute only moderately to the overall binding affinity of the small molecule. A variety of novel substituents were introduced at this position (data not shown), most of which were reasonably well tolerated. However, only groups that improved on the simple compound ***12*** were considered as advanceable. A subset of the more potent analogues exploring this position are shown in Figure 5. Heterocycles appear to be the most advantageous substitution at this position with several examples such as ***13–15*** reaching parity with the parent ***5c*** on potency. Of note, benzenesulfonamide and tetrahydropyran derivatives ***16*** and ***17***, were also amongst the most potent analogues. These results indicate that this position can be further optimised to enhance the ligand efficiency of this series. Parallel work (Shan et al., 2021) reported a significant improvement in activity through modification of the benzylic group (***18*** in Supplementary Figure S7). We attempted to replicate these results (Supplementary Figure S7A) (Shan et al., 2021) and in our hands, this compound is on par with ***5c***, and does not show vastly improved potency (Supplementary Figures S7A,B).

Compound ***9*** (Figure 5) retained high activity (760 nM, comparable to ***5c***) and thus was selected for further orthogonal SPR assays against our panel of DUBs (see above, Table 2 **,** Supplementary Figure S8). The key difference in ***9*** is a substitution on the naphthyl ring, a methoxy group in the 4 position, which does not impact compound potency or binding affinity. This substituent may however modulate positively the properties of the naphthyl ring, which prompted us to perform initial *in vitro* ADME studies on compound ***9***, and compare this with ***3k*** and ***5c***.

#### Preliminary ADME Assessment With Selected PLpro Inhibitors

**FIGURE 6:**
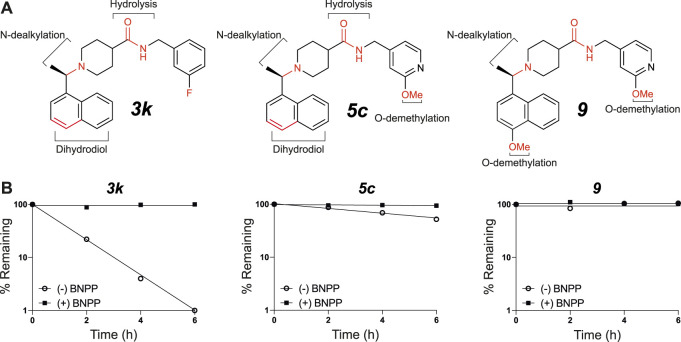
*In vitro* microsomal stability and metabolite studies on compounds ***3k*, *5c*** and ***9***. **(A)** Primary sites of metabolism are indicated following incubation with liver microsomes and **(B)** degradation following incubation with mouse plasma in the absence or presence of the carboxylesterase inhibitor, BNPP (mean of *n* = 2 per time point). In addition to metabolites shown, several mono- and di-oxygenation products were also observed for each compound however sites of oxygenation were not determined.

To obtain an initial assessment of the ADME properties, selected compounds were characterized for their metabolic stability when incubated with human or mouse liver microsomes (HLM or MLM, respectively), mouse plasma stability, kinetic solubility, Caco-2 permeability, and mouse plasma protein binding. As shown in Table 3, both ***3k*** and ***5c*** were metabolically labile in both HLM and MLM. Compound ***9*** was slightly more stable in comparison to ***3k*** and ***5c***. Preliminary metabolite identification studies suggested that common primary metabolites **(** Figure 6A **)** included a dihydrodiol on the naphthalene (confirmed by analysis of the CID spectrum, data not shown), N-dealkylation at the piperidine nitrogen, amide hydrolysis (***3k*** and ***5c***), and O-demethylation (***5c*** and ***9***). Multiple mono- and di-oxygenation products were also detected for each compound, but the sites of oxygenation were not determined.

**TABLE 3:** ADME properties for selected compounds. Microsomal stability.

Compound	cLogD_7.4_	HLM		MLM	
		CL_int_ (µL/min/mg)	T_1/2_ (min)	CL_int_ (µL/min/mg)	T_1/2_ (min)
***3k***	2.5	247^a^	7	337^a^	5
***5c***	1.6	110^a^	16	86^a^	20
***9***	1.7	45	39	50	35

cLog D_7.4_ determined using Jchem for Excel (ChemAxon, ver 21.2.0).

CL_int_, intrinsic clearance.

a Degradation also detected in controls in the absence of cofactor.

Compound ***3k*** was also highly susceptible to hydrolysis in mouse plasma and degradation was prevented by the addition of bis-*para*-nitrophenylphosphate (BNPP), a known inhibitor of carboxylesterases that are present in plasma and various tissues (Eng et al., 2010). Plasma-mediated hydrolysis was also evident for ***5c***, although the rate of degradation was much less pronounced than for ***3k*** (Figure 6B). For both ***3k*** and ***5c***, the amide hydrolysis product was detected at the end of the incubation. Interestingly, hydrolysis of ***9*** was not detected in either microsomes or plasma. Collectively, these results suggest that the *O*-methoxy pyridine (***5c***) in place of the fluorophenyl (***3k***) reduces the rate of hydrolysis (possibly due to a reduction in Log D), and that the combination of the *O*-methoxy pyridine (in both ***5c*** and ***9***) and the methoxy on the naphthalene in ***9*** greatly reduces the rate of hydrolysis of the central amide (Figure 6B).

Kinetic solubility was good (>100 μg/ml) under conditions representative of the fasted gastric environment (pH 2) but was reduced under conditions that reflect the fasted upper small intestine (pH 6.5) where most drug absorption occurs (Table 4). For ***5c***, ***3k*** and ***9***, Caco-2 permeability was high and there was no evidence of efflux suggesting that permeability would not limit oral absorption (Table 4). Mouse plasma protein binding was moderate (***5c*** and ***9***) to high (***3k***) (Table 4).

**TABLE 4:** Kinetic solubility, Caco-2 permeability and mouse plasma protein binding.

Compound	Kinetic solubility (µg/ml)		Caco-2 A-B/B-A P_app_ (10^−6^ cm/s)	Mouse plasma protein binding (% bound)^a^
	pH 2.0	pH 6.5		
***3k***	>100	12.5 – 25	35/36	95.3
***5c***	>100	50 – 100	53/55	86.3
***9***	>140	70 – 140	46/46	90.1

NT, not tested.

a ^Measured in the presence of BNPP, to prevent hydrolysis.

These preliminary ADME results suggest that the major limiting factor for this series to achieve high and prolonged *in vivo* exposure is likely to be hepatic metabolism, accompanied by plasma-mediated hydrolysis for some compounds. Such liabilities are common for early-stage inhibitors and may be addressed by future rounds of medicinal chemistry.

#### 5c Derivatives Inhibitors are Potent Inhibitors of SARS-CoV-2 Infection in Cells

**FIGURE 7:**
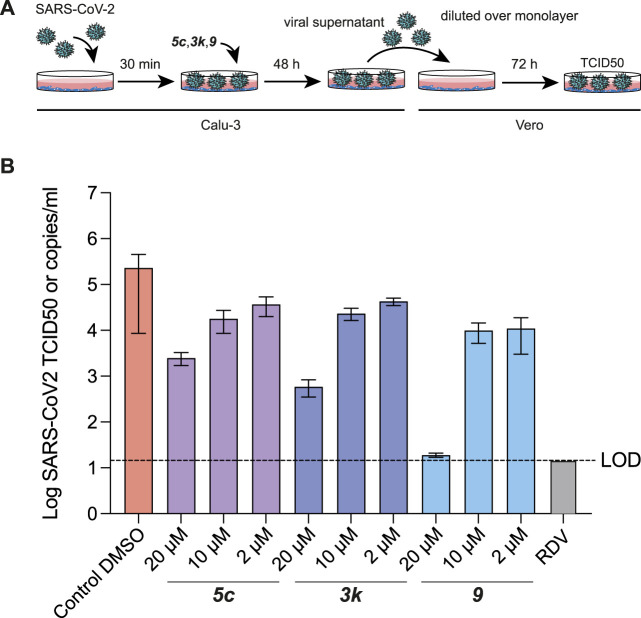
TCID50 Assays for compound ***5c***, ***3k*** and ***9***. **(A)** Schematic and time-course of TCID50 determination. Vero cells were infected with SARS-CoV-2 containing supernatant obtained from infected Calu-3 cells, and treated as shown in the cartoon (see Methods). **(B)** TCID50 data, mean and standard deviation for one of two representative experiments with six technical replicates each. Compound ***9*** retained antiviral activity in preventing viral replication, and in stabilising the naphthyl ring appears to correlate with a modest increase in antiviral activity. Remdesivir (RDV) was used for comparison and assayed at 12.5 µM. Experiments were performed as three independent experiments (*n* = 3) each containing six technical replicates. Values shown are the mean of the three independent experiments, error bars indicate the standard error of the mean. LOD = limit of detection.

Our previous work had already indicated that ***rac5c*** was a potent inhibitor of SARS-CoV-2 replication, in a live-infection model using Calu-3 cells, with no evidence of cytotoxicity up to 33 µM (Klemm et al., 2020). We performed similar assays measuring the median tissue culture infection dose (TCID50), of SARS-CoV-2 infection in presence of increasing amounts of ***5c, 3k*** and ***9*** (Figure 7). Our results again indicated a 1-log reduction of viral titer at 10 µM compound concentration, while 20 µM of compound ***9*** reduced viral titer below the limit of detection (Figure 7B). Overall, these results show again that specific inhibition of SARS-CoV-2 PLpro invokes a potent antiviral response, and that small molecule inhibitors of PLpro may prove to be efficacious as novel COVID-19 treatments.

## Discussion and Conclusion

We here confirm that PLpro is a promising drug target for COVID-19 that requires *de novo* drug discovery. There is currently little evidence that drug repurposing, a method hoped to be a silver bullet to tackle the COVID-19 pandemic, will be of any benefit, and we show in this work and our associated Review that drug repurposing has failed in the context of PLpro as it has in other settings.

Nonetheless, we also provide promising new insights into how piperidine-based PLpro inhibitors, including the well-studied SARS-CoV PLpro inhibitor ***5c***, may be derivatized to generate potent and importantly, more drug-like inhibitors. Indeed, we show that commonly used PLpro inhibitors suffer from a multitude of liabilities, mostly due to the presence of a naphthyl moiety that is present in both ***5c*** but also ***GRL-0617*** compounds (see associated Review). In the context of the ***GRL-0617*** series, we note that recent reports have shown that this moiety can be successfully replaced using a substituted 2-phenylthiophene scaffold with no loss in potency (Shen et al., 2021). Our work shows small changes can also ameliorate the properties of the naphthyl-based inhibitors though it is likely that the path towards *in vivo-* or even clinically suitable compounds is likely to be long.

The druggability of PLpro has so far proven challenging, and high affinity (below 100 nM) compounds have not been reported. Despite this, the considerable efforts applied to inhibiting PLpro and full structural enablement have significantly advanced our understanding such that it remains a viable drug target for treating COVID-19.

Once successful, we anticipate that a PLpro inhibitor will have similarly or even more potent anti-CoV activity, as observed for Mpro inhibitors that have recently been approved. Indeed, in addition to blocking the essential protein processing steps in the viral replication cycle, inhibiting PLpro may also serve additional purposes: as a DUB and deISGylase, PLpro prevents virus-induced derailing of the cellular inflammatory and antiviral cascades affected by PLpro mediated cleavage of ubiquitin and ISG15, and may at least in part be responsible for the observed inflammatory flares reported in COVID-19 patients. We therefore consider PLpro as the ultimate drug target in Coronaviruses, that, although challenging, will likely provide significant safeguarding against future pandemics.

## Methods

### Protein Biochemistry, Structural Biology and Compound Screening

#### Molecular Biology

Bacterial pOPIN-B expression vectors (Berrow et al., 2007) for SARS-CoV-2 PLpro amino acids (aa) 1563-1878 of polyprotein 1 ab, GenBank: QHD43415, with aa E1564 designated as residue 1, were reported previously (Klemm et al., 2020). SARS-CoV PLpro^WT^ (aa 1541-1855 of polyprotein 1 ab, RefSeq: NP_828849.7) and MERS-CoV PLpro^WT^ (aa 1482-1803 of polyprotein 1 ab, RefSeq: YP_009047202) were codon optimised for bacterial expression, synthesized (Integrated DNA Technologies) and cloned into pOPIN-B digested with KpnI and HindIII using In-Fusion® HD cloning (Takara Clontech). The SARS-CoV-2 PLpro BL2 mutant (SARS-CoV-2 PLpro^BL^) was generated by NEB Q5® Site-Directed Mutagenesis of the SARS-CoV-2 PLpro^WT^ plasmid (fwd 5′-GAG​TAT​ACG​GGC​ATC​GAG​ACT​GCA​GTC​GGT​CAC​TAC​AAA C-3′, rev 5′-CGA​TGC​GCA​GGT​GAA​CGT​TC-3′).

For crystallography, we matched a construct used previously (Osipiuk et al., 2021), which has a 1-aa shorter SARS-CoV-2 PLpro sequence (aa 1564-1878) preceded by a Ser-Asn-Ala sequence and includes a catalytic Cys111 mutation to Ser (SARS-CoV-2 PLpro^C111S^). The coding sequence was cloned into pOPIN-S which features a His-SUMO-tag. SUMO protease (SENP1) was produced according to (Pruneda et al., 2016).

#### Protein Purification

SARS-CoV-2 PLpro^WT^, SARS-CoV-2 PLpro^BL^, SARS-CoV PLpro^WT^, MERS-CoV PLpro^WT^ and SARS-CoV-2 PLpro^C111S^ expression vectors were transformed into *E. coli* Rosetta® 2 (DE3) competent cells (Novagen) and bacterial cells were grown in 2xYT medium at 37°C. At OD_600_ = 0.8 the temperature was reduced to 18°C and expression was induced with 0.3 mM IPTG. Cells were harvested 16 h post induction and stored at −80°C until purification.

For purification, cells were resuspended in lysis buffer/Buffer A (50 mM Tris pH 7.5, 500 mM NaCl, 5 mM β-ME, 10 mM Imidazole) supplemented with lysozyme (2 mg/ml), DNaseI (100 μg/ml), MgCl_2_ (5 mM) and cOmplete EDTA-free protease inhibitor cocktail tablets (Roche) and lysed by sonication. Lysates were cleared by centrifugation at 40,000 *g* for 30 min at 4°C. The clarified lysate was filtered through a 0.45 µm syringe filter and His-tagged proteins were captured using a HisTrap HP column (5 ml, Cytiva). The captured protein was washed with 10 CV of 30 mM imidazole wash buffer (Buffer A+ 10% Buffer B) and eluted using five column volumes of 100% Buffer B (Buffer A+ 300 mM Imidazole). Pooled fractions were desalted into 100% Buffer A using a HiPrep 26/10 Desalting column (Cytiva) and then supplemented with His-3C or His-SENP1 protease for His-tag and His-SUMO tag cleavage respectively. Following overnight incubation at 4°C, the cleaved His-tag, His-SUMO tag and His-tagged proteases were captured using a HisTrap HP column (5 ml, Cytiva). The extracted PLpro found in the flow through was further purified by size exclusion chromatography using a HiLoad 16/600 Superdex 75 pg column (Cytiva) equilibrated with storage buffer (20 mM Tris pH 7.5, 150 mM NaCl, 1 mM TCEP).

For HTS, SARS-CoV-2 PLpro^WT^ was purified as above. For SPR storage buffer, 20 mM Tris pH 7.5 was replaced with 10 mM HEPES pH 7.5, for crystallisation, 150 mM NaCl was replaced with 50 mM NaCl. Protein samples were concentrated, flash frozen in liquid nitrogen and stored at -80°C.

#### SARS-2-CoV-PLpro Activity Assay

Assays were essentially performed as described previously (Klemm et al., 2020). In short, SARS-CoV-2 PLpro activity was monitored in a fluorescence intensity assay using the substrate ubiquitin-Rhodamine110, that only becomes fluorescent on cleavage. For HTS, the assay buffer contained 20 mM Tris (pH 8), 1 mM TCEP, 0.03% BSA and 0.01% Triton-X, for all other assays, 1 mM TCEP was replaced with 1 mM GSH. Experiments were performed in 1536-well black non-binding plates (Greiner 782900) with a final reaction volume of 6 µL.

SARS-2-CoV PLpro enzyme (final concentration 50 nM) was added to the plates and incubated at room temperature for 10 min. ubiquitin-Rhodamine110 (final concentration 100 nM) was added to start the reaction and incubated for 12 min at room temperature. For endpoint assays the reaction was stopped by addition of citric acid (1 µL) at a final concentration of 10 mM. All additions were performed using the CERTUS FLEX (v2.0.1, Gyger). The reaction was monitored by an increase in fluorescence (excitation 485 nm and emission 520 nm) on a PHERAstar® (v5.41, BMG Labtech) using the FI 485 520 optic module.

Data was normalised to 1% DMSO (negative control, 0% inhibition) and 100 µM Compound 5c (positive control, 100% inhibition).

#### SARS-2-CoV-PLpro^WT^ Gel Based Activity Assay

Assays were essentially performed as described previously (Klemm et al., 2020). In short, SARS-2-CoV PLpro activity was monitored using SDS-PAGE and tracking the cleavage of K48 Ub_3_ or hISG15 by SARS-CoV-2 PLpro^WT^ or SARS-CoV-2 PLpro^BL^, over time. Each respective enzyme was incubated at 0.25 µM with 2 µM substrate and the reaction was stopped at indicated time points by mixing with NuPAGE® loading dye supplemented with β-mercaptoethanol. The assay buffer contained 20 mM Tris (pH 7.5), 100 mM NaCl and 10 mM DTT. Experiments were performed at 21°C.

#### SARS-2-CoV-PLpro^WT^ Specificity Assay (Ubiquigent)

SARS-CoV-2 PLpro^WT^ protein and compound ***5c*** were supplied to Ubiquigent (Dundee, United Kingdom). ***5c*** was assayed using the commercial Ubiquigent Ub-Rh based DUBprofiler® drug discovery screening platform and results were analysed and provided by Ubiquigent.

#### High Throughput Screen of the ReFRAME Library

A total of 11,804 compounds from the ReFrame (Repurposing, Focused Rescue and Accelerated Medchem) library were screened. Assay-ready plates were prepared at the Global Health Drug Discovery Institute (GHDDI), China. 5 nL of compounds were dry spotted onto 1536-well plates. Stock concentrations of compounds were 10, 5 and 1 mM and final test concentrations were 8.33, 4.17 and 0.83 µM respectively in final 1% DMSO.

Reagents were dispensed using the CERTUS FLEX (v2.0.1, Gyger). Microplates were centrifuged using the Microplate Centrifuge (Agilent) and read on the PHERAstar® (v5.41, BMG Labtech) using the FI 485 520 optic module.

Data was normalised to 1% DMSO (negative control, 0% inhibition) and 100 µM Compound 5c (positive control, 100% inhibition). Screen assay quality was monitored by calculation of robust Z’ by the following formula where (+) denotes the positive controls (low signal), (-) denotes the negative controls (high signal) and MAD is the median absolute deviation:

$$\text{robust Z' }=\text{ 1}-\;\left(3*\left({\text{MAD}}_{-}{\;+\text{ MAD}}_{+}\right)/\text{abs}\left({\text{median}}_{-}{\;-\text{ median}}_{+}\right)\right)$$

where MAD = 1.4826 * median (abs (x – median(x)))

Plates were excluded from analysis if robust Z’<0.5. Hits were selected as >2.5* MAD over the median of the negative control.

To determine the potency of the inhibitors, a series of 10-pt, 1:2 serial dilutions was performed from a highest starting concentration of 100 µM. The 10-point titration curves were fitted with a 4-parameter logistic nonlinear regression model and the IC_50_ reported is the inflection point of the curve. Data was analysed in TIBCO Spotfire® 7.11.2.

#### Counter Screen

To confirm that the compounds were specifically inhibiting SARS-CoV-2 PLpro rather than interfering with the fluorescence readout, human USP21 was used as the counter screen assay as previously (Klemm et al., 2020). The same buffer, reagent dispenser and plate reader as in the PLpro assay was used. USP21 enzyme (final concentration 5 nM) was added to the plates and incubated at room temperature for 10 min. ubiquitin-Rhodamine110 (final concentration 100 nM) was added to start the reaction and incubated for 2 min at room temperature. Reaction was stopped by addition of citric acid (1 µL) at a final concentration of 10 mM. A series of 10-pt, 1:3 serial dilutions was performed from a highest starting concentration of 100 µM. The 10-point titration curves were fitted with a 4-parameter logistic nonlinear regression model and the IC_50_ reported is the inflection point of the curve. Data was analysed in TIBCO Spotfire® 7.11.2.

#### Crystallisation

Crystallisation screening was performed at the CSIRO’s Collaborative Crystallisation Centre (C3) in Melbourne, Australia. The SARS-CoV-2 PLpro complex with 3k was generated by incubation of SARS-CoV-2 PLpro^C111S^ (13 mg/ml) with 2 mM inhibitor, overnight at 4°C and precipitate removed by centrifugation. Crystals grew in 0.1 M bis-tris chloride pH 5.46, 0.117 M Zinc Acetate, 21.6% PEG 8000 in a 96-well sitting drop vapour diffusion plate (150 nL protein to 150 nL reservoir solution) at 8°C. Crystals were cryoprotected with reservoir solution supplemented with 15% glycerol and 1 mM inhibitor before vitrification in liquid nitrogen.

#### Data Collection, Phasing and Refinement

Diffraction data were collected at the Australian Synchrotron (Australian Nuclear Science and Technology Organisation, ANSTO) beamline MX2 (Aragão et al., 2018) (wavelength: 0.953725 Å, temperature: 100 K). An auto-processed dataset was generated at the synchrotron using XDS, Aimless and Pointless (Evans, 2006, 2011; Kabsch, 2010). The dataset was solved by molecular replacement in Phaser (McCoy et al., 2007) using the apo structure of SARS-CoV-2 PLpro [PDB 6WRH (Osipiuk et al., 2021)] as a search model.

Refinement and model building was performed in PHENIX (Adams et al., 2011) and Coot (Emsley et al., 2010). TLS parameters were set to one TLS group per chain. Additional NCS refinement was utilised in each refinement cycle. Geometric restraints for ***3k*** were generated by the GRADE web server (http://grade.globalphasing.org). Models were validated using MolProbity (Williams et al., 2018). Final Ramachandran statistics were 0.00% outliers, 1.63% allowed and 98.64% favoured. Structural figures were generated using PyMol. Further data collection and refinement statistics can be found in Table 1.

#### Surface Plasmon Resonance

Experiments were performed on a BIAcore 8K + instrument (Cytiva, United States) PLpro proteins were diluted into 10 mM sodium acetate pH 5 prior to immobilisation on a CM5 sensor chip (Cytiva, United States) by amine coupling. Compounds were diluted to desired concentrations between 20 and 0.01 µM in a running buffer consisting of 20 mM HEPES pH 7.4, 150 mM sodium chloride, 0.05% P20 detergent,1 mM TCEP and 2% DMSO. Multi cycle kinetics were performed with 90 s associations and 300 s dissociations with no further regeneration. Binding constants were determined in BIAcore insight evaluation (version 3.0.12) at steady-state, averaging response over 5 s with a midpoint 5 s before the end of the association phase. Final K_D_ values were determined by averaging the values from three independent experiments, reporting mean and standard deviation.

### Medicinal Chemistry

#### Experimental

All reagents were used as received from commercial suppliers unless otherwise stated. NMR spectra were recorded at ambient temperature either on Bruker Avance II^TM^ 300 MHz, Bruker Avance III^TM^ 400 MHz or Bruker Avance III^TM^ HD 400 MHz instruments in the specified deuterated solvents. Observed proton absorptions were reported as units of parts per million (ppm) relative to respective residual solvent peaks, CDCl_3_ (d 7.26), DMSO-d6 (d 2.50). Multiplicities were reported: s (singlet), d (doublet), t (triplet), q (quartet), dd (doublet of doublets), dt (doublet of triplets) and m (multiplet). Coupling constants were reported as a J value in Hertz (Hz). HPLC/UPLC and LC-MS data was obtained on either an Agilent 6120 series with a Phenomenex Poroshell 120 EC-C18, (2.1 mm × 30 mm, 2.7 mm) column^#^ or Waters Acquity H-Class UPLC/MS with Acquity HSS-T3 (2.1 mm × 100 mm, 1.8 mm) column^*^ or Prontosil-EP1 (4.6 × 250 mm) 5 μm column^^^ using a gradient elution of 5–100% acetonitrile in water containing 0.1% formic acid. Preparative HPLC was performed on a Waters X-Bridge TM prep C18 OBD column (19 mm × 100 mm, 5 mM) using various gradients based on analytical retentions using water and acetonitrile containing 0.1% formic acid over 10 min at a flow rate of 20 ml/min. Abbreviations: DCM (dichloromethane), EDCI[N-(3-Dimethylaminopropyl)-N′-ethylcarbodiimide hydrochloride], DIEA (N,N-diisopropylamine), THF (tetrahydrofuran), MeOH (methanol), EtOAc (ethyl acetate), EtOH (ethanol), DMF (N,N′-dimethylformamide), HATU {1-[Bis(dimethylamino)methylene]-1*H*-1,2,3-triazolo [4,5-*b*]pyridinium 3-oxid hexafluorophosphate}.

Literature compounds ***3k*** (Báez-Santos et al., 2014), ***5c*** (Báez-Santos et al., 2014) and ***18*** (Shan et al., 2021) were synthesised as previously described in their respective references.

#### 3k

^1^H NMR (300 MHz, CDCl_3_) δ 8.48 – 8.38 (m, 1H), 7.90 – 7.80 (m, 1H), 7.74 (d, *J* = 8.0 Hz, 1H), 7.57 (d, *J* = 7.3 Hz, 1H), 7.53 – 7.38 (m, 3H), 7.32 – 7.22 (m, 1H), 7.01 (d, *J* = 7.5 Hz, 1H), 6.99 – 6.90 (m, 2H), 5.76 (s, 1H), 4.43 (d, *J* = 5.8 Hz, 2H), 4.11 (q, *J* = 6.3 Hz, 1H), 3.24 (d, *J* = 11.0 Hz, 1H), 2.90 (d, *J* = 11.6 Hz, 1H), 2.22 – 1.64 (m, 7H), 1.47 (d, *J* = 6.7 Hz, 3H). ES + MS: (M + H) 391.2. HPLC^#^ *t*_g_ = 1.59 min.

#### 5c

^1^H NMR (300 MHz, CDCl_3_) δ 8.48 – 8.39 (m, 1H), 8.08 (dd, *J* = 5.3, 0.6 Hz, 1H), 7.89 – 7.80 (m, 1H), 7.74 (d, *J* = 8.1 Hz, 1H), 7.57 (d, *J* = 6.5 Hz, 1H), 7.52 – 7.38 (m, 3H), 6.73 (dd, *J* = 5.3, 1.4 Hz, 1H), 6.58 (dd, *J* = 1.4, 0.7 Hz, 1H), 5.79 (t, *J* = 5.7 Hz, 1H), 4.39 (d, *J* = 6.0 Hz, 2H), 4.11 (q, *J* = 6.6 Hz, 1H), 3.91 (s, *J* = 2.8 Hz, 3H), 3.24 (d, *J* = 11.7 Hz, 1H), 2.91 (d, *J* = 11.3 Hz, 1H), 2.24 – 1.68 (m, 7H), 1.47 (d, *J* = 6.7 Hz, 3H). ES + MS: (M + H) 404.2. HPLC^#^ *t*_g_ = 1.23 min.

#### 18

^1^H NMR (300 MHz, DMSO) δ 8.70 (s, 1H), 8.50 – 8.40 (m, 1H), 8.27 (t, *J* = 5.6 Hz, 1H), 7.98 – 7.86 (m, 1H), 7.80 (d, *J* = 7.9 Hz, 1H), 7.63 – 7.40 (m, 4H), 7.28 (dt, *J* = 12.0, 2.3 Hz, 1H), 7.11 (s, 1H), 6.63 – 6.49 (m, 1H), 4.27 – 4.03 (m, 3H), 3.48 – 3.38 (m, 4H), 3.07 (s, 1H), 2.78 (s, 1H), 2.33 (s, 4H), 2.25 – 2.08 (m, 4H), 2.01 (s, 2H), 1.73 (d, *J* = 12.0 Hz, 1H), 1.67 – 1.47 (m, 3H), 1.41 (d, *J* = 6.0 Hz, 3H). ES + MS: (M + H) 532.0 HPLC^#^ *t*_g_ = 0.99 min.

#### General Methods – Compounds 1–9


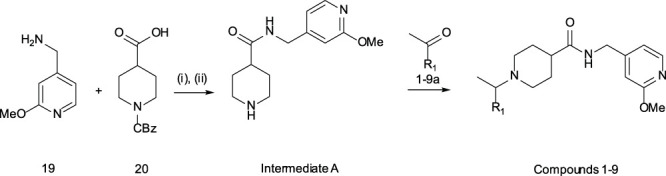


Step (i): To a stirred solution of 1-[((benzyloxy)carbonyl]piperidine-4-carboxylic acid (3.00 g, 11.4 mmol) in DCM (60 ml) was added EDCI (5.46 g, 28.5 mmol), 1H-1,2,3-benzotriazol-1-ol hydrate (4.36 g, 28.5 mmol) and DIEA (9.95 ml, 57.0 mmol). After stirring for 10 min, (2-methoxypyridin-4-yl)methanamine (1.89 g, 13.7 mmol) was added under N_2_ atmosphere. The reaction mixture was stirred at ambient temperature until completion of the reaction (TLC monitoring), the reaction was quenched with saturated NH_4_Cl (50 ml) and extracted with DCM (2 × 50 ml). The combined organic layer was dried over anhydrous Na_2_SO_4_ and evaporated under reduced pressure. The compound was purified by CombiFlash (SiO_2_, 100% EtOAc) to give benzyl 4-{[(2-methoxypyridin-4-yl)methyl]carbamoyl}piperidine-1-carboxylate (2.80 g, 64% yield) as an off-white solid. ^1^H NMR (400 MHz, DMSO) δ 8.43 (t, *J* = 5.7 Hz, 1H), 8.07 (d, *J* = 5.2 Hz, 1H), 7.44 – 7.26 (m, 5H), 6.82 (d, *J* = 5.0 Hz, 1H), 6.61 (s, 1H), 5.07 (s, 2H), 4.23 (d, *J* = 5.9 Hz, 2H), 4.02 (d, *J* = 13.2 Hz, 2H), 3.82 (s, 3H), 2.84 (br s, 2H), 2.47 – 2.36 (m, 1H), 1.74 (d, *J* = 11.5 Hz, 2H), 1.47 (qd, *J* = 12.5, 4.2 Hz, 2H). ES + MS: (M + H) 384.15 HPLC^*^ *t*_g_ = 2.08 min.

Step (ii): To a stirred solution of benzyl 4-{[(2-methoxypyridin-4-yl)methyl]carbamoyl}piperidine-1-carboxylate (2.80 g, 7.30 mmol) in THF (20 ml) and MeOH (20 ml) was added 20% Pd(OH)_2_ on carbon (2.80 g, 100% w/w) at ambient temperature. The resulting mixture was stirred for 3 h under H_2_ pressure (atm). After completion of reaction (by TLC monitoring) the reaction mixture was filtered through Celite® and the filter cake washed with MeOH. The filtrate was collected and concentrated *in vacuo* to give **Intermediate A** as a yellow oil. The product was used without purification for further reaction. ^1^H NMR (400 MHz, DMSO) δ 8.35 (s, 1H), 8.07 (d, *J* = 5.3 Hz, 1H), 6.82 (d, *J* = 5.2 Hz, 1H), 6.59 (s, 1H), 4.22 (d, *J* = 6.0 Hz, 2H), 3.82 (s, 3H), 2.99 (d, *J* = 12.3 Hz, 2H), 2.76 (d, *J* = 11.7 Hz, 1H), 2.37 – 2.24 (m, 1H), 2.20 – 2.04 (m, 1H), 1.92 – 1.76 (m, 1H), 1.74 – 1.38 (m, 4H). ES + MS: (M + H) 250.15 HPLC^*^ *t*_g_ = 0.58 min.

#### General Reductive Alkylation

*1-*{*1-[(1,1′-biphenyl*)*-3-yl*]*ethyl*}*-N-*[(*2-methoxypyridin-4-yl*)*methyl*]*piperidine-4-carboxamide*, **(*1*)**. To a stirred solution of 1-[(1,1′-biphenyl)-3-yl]ethenone (133 mg, 0.68 mmol) and Intermediate A (170 mg, 0.68 mmol) in THF (10 ml) at 0°C was added Ti(O^*i*^Pr)_4_ (621 μL, 2.05 mmol) under nitrogen and the temperature raised to 80°C for 16 h. The reaction mixture was cooled to 0°C, diluted with MeOH (5 ml) and then sodium borohydride (61.0 mg, 1.70 mmol) was added under N_2_. The reaction mixture was allowed to achieve ambient temperature and stirred until complete by LCMS & TLC. The reaction mixture was concentrated in vacuo and diluted with saturated NaHCO_3_ (10 ml) and extracted with EtOAc (3 × 10 ml). The combined organics were dried over anhydrous Na_2_SO_4_ and concentrated *in vacuo*. The crude residue was purified by preparative HPLC to give the title compound (138 mg, 47% yield). ^1^H NMR (300 MHz, DMSO) δ 8.30 (t, *J* = 6.0 Hz, 1H), 8.15 (s, 1H), 8.05 (d, *J* = 5.7 Hz, 1H), 7.69 – 7.61 (m, 2H), 7.59 – 7.27 (m, 7H), 6.80 (dd, *J* = 5.3, 1.3 Hz, 1H), 6.58 (s, 1H), 4.21 (d, *J* = 5.9 Hz, 2H), 3.81 (s, 3H), 3.57 (q, *J* = 6.7 Hz, 1H), 3.04 (d, *J* = 11.1 Hz, 1H), 2.85 (d, *J* = 11.5 Hz, 1H), 2.21 – 2.06 (m, 1H), 2.05 – 1.84 (m, 2H), 1.81 – 1.48 (m, 4H), 1.35 (d, *J* = 6.8 Hz, 3H). ES + MS: (M + H) 430.2 HPLC^#^ *t*_g_ = 1.36 min.

*1-*{*1-*[*benzo(b)thiophen-3-yl*]*ethyl*}*-N-*[(*2-methoxypyridin-4-yl*)*methyl*]*piperidine-4-carboxamide formate* **(*2*)**. The title compound was prepared as described for compound ***1*** from 1-[benzo(b)thiophen-3-yl]ethanone in 20% yield. ^1^H NMR (400 MHz, DMSO) δ 8.30 (t, *J* = 6.0 Hz, 1H), 8.13 (dd, *J* = 9.4, 8.0 Hz, 2H), 8.05 (d, *J* = 5.2 Hz, 1H), 7.98 – 7.92 (m, 1H), 7.52 (s, 1H), 7.36 (pd, *J* = 7.0, 1.2 Hz, 2H), 6.79 (d, *J* = 4.2 Hz, 1H), 6.57 (s, 1H), 4.20 (d, *J* = 5.9 Hz, 2H), 4.04 (q, *J* = 6.5 Hz, 1H), 3.80 (s, 3H), 2.95 – 2.81 (m, 2H), 2.18 – 2.05 (m, 2H), 1.97 (t, *J* = 10.4 Hz, 1H), 1.73 – 1.54 (m, 3H), 1.53 – 1.36 (m, 4H). ES + MS: (M + H) 410.17 HPLC* *t*_g_ = 4.59 min.

*N-*[(*2-methoxypyridin-4-yl*)*methyl*]*-1-*{*1-*[*2-methylbenzo(b)thiophen-3-yl*]*ethyl*}*piperidine-4-carboxamide* **(*3*)**. The title compound was prepared as described for compound ***1*** from 1-[2-methylbenzo (b)thiophen-3-yl]ethanone in 2% yield. ^1^H NMR (400 MHz, DMSO) δ 8.34 (t, *J* = 6.0 Hz, 1H), 8.22 (d, *J* = 8.0 Hz, 1H), 8.05 (d, *J* = 5.2 Hz, 1H), 7.82 (d, *J* = 7.7 Hz, 1H), 7.33 – 7.23 (m, 2H), 6.81 (d, *J* = 5.0 Hz, 1H), 6.59 (s, 1H), 4.21 (d, *J* = 6.0 Hz, 2H), 3.81 (s, 3H), 3.65 (q, *J* = 6.7 Hz, 1H), 3.34 (d, *J* = 8.0 Hz, 1H), 3.29 (s, 3H), 2.60 (d, *J* = 11.3 Hz, 1H), 2.22 – 2.14 (m, 1H), 1.95 – 1.64 (m, 4H), 1.55 (d, *J* = 11.7 Hz, 1H), 1.50 – 1.33 (m, 4H). ES + MS: (M + H) 424.03 HPLC* *t*_g_ = 6.31 min.

*1-*(*1-*(*5-chloro-2-methoxyphenyl*)*ethyl*)*-N-*((*2-methoxypyridin-4-yl*)*methyl*)*piperidine-4-carboxamide* **(*4*)**. The title compound was prepared as described for compound ***1*** from 1-(5-chloro-2-methoxyphenyl)ethanone in 82% yield. ^1^H NMR (300 MHz, Chloroform-d) δ 8.08 (dd, J = 5.3, 0.7 Hz, 1H), 7.41 (d, J = 2.7 Hz, 1H), 7.14 (dd, J = 8.7, 2.7 Hz, 1H), 6.82 – 6.70 (m, 2H), 6.58 (h, J = 1.5, 0.8 Hz, 1H), 5.85 (s, 1H), 4.40 (d, J = 6.1 Hz, 2H), 3.91 (s, 4H), 3.79 (s, 3H), 3.21 (d, J = 11.2 Hz, 1H), 2.87 (d, J = 9.3 Hz, 1H), 2.22 – 1.65 (m, 7H), 1.26 (d, J = 6.7 Hz, 3H). ES + MS: (M + H) 418.0 HPLC^#^ *t*_g_ = 1.09 min.

*1-*[*1-*(*5-chloro-2-ethoxyphenyl*)*ethyl*]*-N-*[(*2-methoxypyridin-4-yl*)*methyl*]*piperidine-4-carboxamide* **(*5*)**. The title compound was prepared as described for compound ***1*** from 1-(5-chloro-2-ethoxyphenyl)ethanone in 65% yield. ^1^H NMR (300 MHz, Chloroform-d) δ 8.08 (dd, J = 5.3, 0.7 Hz, 1H), 7.39 (d, J = 2.7 Hz, 1H), 7.12 (dd, J = 8.7, 2.7 Hz, 1H), 6.80 – 6.70 (m, 2H), 6.62 – 6.55 (m, 1H), 5.84 (s, 1H), 4.40 (d, J = 6.0 Hz, 2H), 4.05 – 3.88 (m, 6H), 3.20 (d, J = 11.0 Hz, 1H), 2.89 (d, J = 11.1 Hz, 1H), 2.23 – 1.67 (m, 7H), 1.40 (t, J = 7.0 Hz, 3H), 1.27 (d, J = 6.8 Hz, 3H). ES + MS: (M + H) 432.2 HPLC^#^ *t*_g_ = 1.28 min.

*1-*[*1-*(*2-methoxynaphthalen-1-yl*)*ethyl*]*-N-*[(*2-methoxypyridin-4-yl*)*methyl*]*piperidine-4-carboxamide formate* **(*6*)**. The title compound was prepared as described for compound ***1*** from 1-(2-methoxynaphthalen-1-yl)ethanone in 15% yield. ^1^H NMR (400 MHz, DMSO) δ 9.01 (d, *J* = 7.3 Hz, 1H), 8.34 (t, *J* = 5.6 Hz, 1H), 8.15 (s, 1H), 8.05 (d, *J* = 5.2 Hz, 1H), 7.82 (d, *J* = 8.7 Hz, 2H), 7.43 – 7.39 (m, 2H), 7.32 (t, *J* = 7.5 Hz, 1H), 6.80 (d, *J* = 5.2 Hz, 1H), 6.58 (s, 1H), 4.35 (d, *J* = 4.9 Hz, 1H), 4.20 (d, *J* = 5.8 Hz, 2H), 3.89 (s, 3H), 3.80 (s, 3H), 3.44 (d, *J* = 10.7 Hz, 1H), 2.55 (d, *J* = 10.9 Hz, 1H), 2.24 – 2.13 (m, 1H), 1.93 (s, br, 1H), 1.88 – 1.70 (m, 3H), 1.50 (d, *J* = 13.7 Hz, 1H), 1.46 – 1.34 (m, 4H). ES + MS: (M + H) 434.25 HPLC* *t*_g_ = 4.62 min.

*1-*[*1-*(*2-ethoxynaphthalen-1-yl*)*ethyl*]*-N-*[(*2-methoxypyridin-4-yl*)*methyl*]*piperidine-4-carboxamide* **(*7*)**. The title compound was prepared as described for compound ***1*** from 1-(2-ethoxynaphthalen-1-yl)ethanone in 4% yield. ^1^H NMR (400 MHz, DMSO) δ 9.03 (d, *J* = 8.6 Hz, 1H), 8.33 (t, *J* = 5.9 Hz, 1H), 8.05 (d, *J* = 5.2 Hz, 1H), 7.79 (t, *J* = 9.6, 2H), 7.46 – 7.28 (m, 3H), 6.80 (d, *J* = 5.1 Hz, 1H), 6.58 (s, 1H), 4.36 (q, *J* = 6.5 Hz, 1H), 4.25 – 4.09 (m, 4H), 3.80 (s, 3H), 3.42 (d, *J* = 11.2 Hz, 1H), 2.55 (d, *J* = 12.3 Hz, 1H), 2.24 – 2.12 (m, 1H), 1.89 (t, *J* = 10.7 Hz, 1H), 1.85 – 1.67 (m, 3H), 1.49 (d, *J* = 12.4 Hz, 1H), 1.45 – 1.32 (m, 7H). ES + MS: (M + H) 448.22 HPLC* *t*_g_ = 4.87 min.

*1-*[*1-*(*4-fluoronaphthalen-1-yl*)*ethyl*]*-N-*[(*2-methoxypyridin-4-yl*)*methyl*]*piperidine-4-carboxamide* **(*8*)**. The title compound was prepared as described for compound ***1*** from 1-(4-fluoronaphthalen-1-yl)ethanone in 48% yield. ^1^H NMR (400 MHz, DMSO) δ 8.51 (d, *J* = 7.7 Hz, 1H), 8.32 (t, *J* = 5.8 Hz, 1H), 8.06 (t, *J* = 5.6 Hz, 2H), 7.67 – 7.58 (m, 2H), 7.51 (dd, *J* = 7.6, 6.0 Hz, 1H), 7.27 (dd, *J* = 10.4, 8.2 Hz, 1H), 6.80 (d, *J* = 5.1 Hz, 1H), 6.57 (s, 1H), 4.20 (d, *J* = 5.9 Hz, 2H), 4.15 (q, *J* = 6.5 Hz, 1H), 3.80 (s, 3H), 3.02 (d, *J* = 10.8 Hz, 1H), 2.80 (d, *J* = 11.2 Hz, 1H), 2.22 – 2.10 (m, 1H), 2.09 – 1.96 (m, 2H), 1.71 (d, *J* = 12.4 Hz, 1H), 1.67 – 1.44 (m, 3H), 1.39 (d, *J* = 6.6 Hz, 3H). ES + MS: (M + H) 422.21 HPLC* *t*_g_ = 5.88 min.

*1-*[*1-*(*4-methoxynaphthalen-1-yl*)*ethyl*]*-N-*[(*2-methoxypyridin-4-yl*)*methyl*]*piperidine-4-carboxamide* **(*9*)**. The title compound was prepared as described for compound ***1*** from 1-(4-methoxynaphthalen-1-yl)ethanone in 17% yield. ^1^H NMR (400 MHz, DMSO) δ 8.41 (d, *J* = 8.3 Hz, 1H), 8.32 (t, *J* = 5.8 Hz, 1H), 8.17 (d, *J* = 8.0 Hz, 1H), 8.05 (d, *J* = 5.3 Hz, 1H), 7.50 (dt, *J* = 14.8, 7.1 Hz, 2H), 7.42 (d, *J* = 8.0 Hz, 1H), 6.91 (d, *J* = 8.1 Hz, 1H), 6.80 (d, *J* = 5.3 Hz, 1H), 6.57 (s, 1H), 4.20 (d, *J* = 5.8 Hz, 2H), 4.12 – 4.01 (m, 1H), 3.95 (s, 3H), 3.80 (s, 3H), 3.02 (d, *J* = 10.2 Hz, 1H), 2.82 (d, *J* = 11.2 Hz, 1H), 2.23 – 2.08 (m, 2H), 2.01 (q, *J* = 10.0 Hz, 2H), 1.70 (d, *J* = 13.3 Hz, 1H), 1.66 – 1.45 (m, 2H), 1.38 (d, *J* = 6.5 Hz, 3H). ES + MS: (M + H) 434.21 HPLC* *t*_g_ = 4.72 min.

#### General Methods – Compound 10


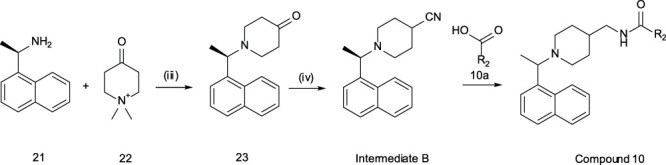


Step (iii): (1R)-1-(1-naphthyl)ethanamine (1.41 g, 8.24 mmol) and potassium carbonate (2.56 g, 18.3 mmol) were taken up in EtOH (20 ml) and Water (5 ml) and warmed to 60^o^C. Concurrently, 1,1-dimethylpiperidin-1-ium-4-one iodide (2.31 g, 9.06 mmol) was dissolved in EtOH:water (1:2, 5 ml) and added drop-wise to the previous mixture. The resulting mixture was heated to reflux for 4 h. After this time the EtOH was removed *in vacuo* and the remaining aqueous extracted with EtOAc. The extracts were combined, dried over anhydrous MgSO_4_, filtered, and concentrated in vacuo. The crude material was purified by combi-flash (SiO_2_, 0–15%EtOAc/DCM) to give 1-[(1R)-1-(1-naphthyl)ethyl]piperidin-4-one (1.44 mg, 69% yield) as a yellow oil. ^1^H NMR (300 MHz, CDCl_3_) δ 8.50 – 8.41 (m, 1H), 7.92 – 7.83 (m, 1H), 7.77 (d, *J* = 8.2 Hz, 1H), 7.62 (d, *J* = 7.1 Hz, 1H), 7.56 – 7.41 (m, 3H), 4.34 (q, *J* = 6.7 Hz, 1H), 2.98 – 2.73 (m, 4H), 2.52 – 2.32 (m, 4H), 1.54 (d, *J* = 6.7 Hz, 3H). ES + MS: (M + H) 272.1. HPLC^#^ *t*_g_ = 0.89 min.

Step (iv): To a solution of 1-[(1R)-1-(1-naphthyl)ethyl]piperidin-4-one (1.43 g, 5.65 mmol) in THF (40 ml) was added 1-(isocyanomethylsulfonyl)-4-methyl-benzene (1.21 g, 6.17 mmol) followed by potassium tert-butoxide (760 mg, 6.77 mmol) and dry MeOH (5 ml). The reaction was stirred at ambient temperature over 18 h. After this time the reaction was concentrated in vacuo and the crude residue partitioned between saturated NaHCO_3_ and EtOAc. The layers were separated and the aqueous further extracted with EtOAc. The extracts were combined, dried over anhydrous MgSO_4_, filtered, and concentrated in vacuo. The crude residue was purified by combi-flash (SiO_2_, 0–5%EtOAc/DCM) to give 1-[(1R)-1-(1-naphthyl)ethyl]piperidine-4-carbonitrile (417 mg, 28% yield) **INTERMEDIATE B** as a yellow oil. ^1^H NMR (300 MHz, CDCl_3_) δ 8.44 – 8.35 (m, 1H), 7.91 – 7.81 (m, 1H), 7.75 (d, *J* = 8.1 Hz, 1H), 7.57 – 7.39 (m, 5H), 4.16 (q, *J* = 6.7 Hz, 1H), 2.97 – 2.76 (m, 1H), 2.76 – 2.55 (m, 2H), 2.52 – 2.27 (m, 2H), 2.00 – 1.74 (m, 4H), 1.47 (d, *J* = 6.7 Hz, 3H). ES + MS: (M + H) 265.2. HPLC^#^ *t*_g_ = 1.11 min.

*(R)-2-methoxy-N-*((*1-*(*1-*(*naphthalen-1-yl*)*ethyl*)*piperidin-4-yl*)*methyl*)*isonicotinamide* **(*10*)**. To a 0°C solution of INTERMEDIATE B (88.4 mg, 0.334 mmol) in dry THF (3 ml) under N_2_ was added lithium aluminium hydride (14.2 mg, 0.374 mmol) in one portion. The reaction was allowed to achieve ambient temperature and stirred over 18 h. The reaction was quenched by minimal drop-wise addition of 1M Rochelle’s salt. The reaction mixture was then filtered through Celite®, the filter cake washed with EtOAc and the filtrate concentrated in vacuo. The crude material was used directly in the subsequent coupling step without further purification. To a solution of crude amine (45.0 mg, 0.168 mmol), 2-methoxypyridine-4-carboxylic acid (25.7 mg, 0.168 mmol) and DIEA (35.0 µL, 0.201 mmol) in DMF (3 ml) was added HATU (64.0 mg, 0.168 mmol). The reaction was stirred at ambient temperature over 18 h. The reaction was diluted with saturated NaHCO_3_ and extracted with EtOAc. The extracts were combined, washed with water (x3) and brine, dried over anhydrous MgSO_4_, filtered, and concentrated in vacuo. The crude material was purified by preparative HPLC to give 2-methoxy-N-[[1-[(1R)-1-(1-naphthyl)ethyl]-4-piperidyl]methyl]pyridine-4-carboxamide (23.3 mg, 34% yield) as a pale yellow oil. ^1^H NMR (300 MHz, CDCl_3_) δ 8.26 – 8.17 (m, 2H), 8.04 (d, *J* = 8.3 Hz, 1H), 7.98 – 7.88 (m, 2H), 7.83 (s, 1H), 7.66 – 7.50 (m, 3H), 7.39 (dd, *J* = 5.4, 1.5 Hz, 1H), 5.16 – 5.07 (m, 1H), 3.97 (d, *J* = 12.0 Hz, 1H), 3.92 (s, 3H), 3.32 (td, *J* = 13.5, 6.7 Hz, 3H), 2.70 – 2.33 (m, 3H), 2.09 – 1.89 (m, 6H), 1.80 (d, *J* = 11.7 Hz, 1H). ES + MS: (M + H) 404.2. HPLC^#^ *t*_g_ = 1.39 min.

#### General Methods – Compounds 12–17


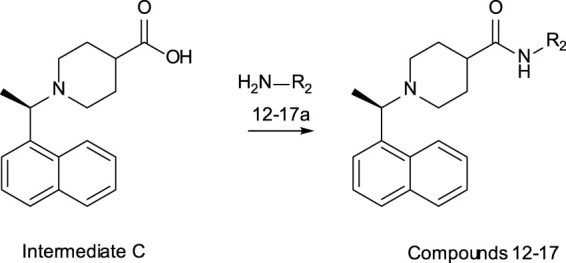


**Intermediate C** was prepared as previously described (Báez-Santos et al., 2014).

^1^H NMR (300 MHz, MeOD) δ 8.30 (d, *J* = 8.5 Hz, 1H), 8.02 (t, *J* = 8.5 Hz, 2H), 7.87 (d, *J* = 7.4 Hz, 1H), 7.74 – 7.56 (m, 3H), 5.45 (dd, *J* = 13.3, 6.6 Hz, 1H), 4.04 (d, *J* = 10.6 Hz, 1H), 3.29 – 3.08 (m, 2H), 2.98 (t, *J* = 13.4 Hz, 1H), 2.59 (t, *J* = 12.5 Hz, 1H), 2.29 (d, *J* = 12.7 Hz, 1H), 2.21 – 1.95 (m, 2H), 1.88 (d, *J* = 6.8 Hz, 3H), 1.83 – 1.65 (m, 1H). ES + MS: (M + H) 284.1. HPLC^*^ *t*_g_ = 1.04 min.

#### General Amide Coupling – Compounds 12–17

*(R)-N-methyl-1-*(*1-*(*naphthalen-1-yl*)*ethyl*)*piperidine-4-carboxamide* **(*12*)**. To a solution of Intermediate C (50 mg, 0.18 mmol) and methylamine hydrochloride (13 mg, 0.19 mmol) in DMF (3 ml) under N_2_ was added DIEA (70 μL, 0.40 mmol) followed by HATU (75 mg, 0.20 mmol). The reaction stirred at ambient temperature until complete by LCMS. The reaction was diluted with water and extracted with EtOAc. The extracts were combined, washed with water (x3) and brine, dried over anhydrous MgSO_4_, filtered and concentrated in vacuo. The crude residue was purified by preparative HPLC to give the title compound in 32% yield. ^1^H NMR (400 MHz, DMSO) δ 8.45 (d, *J* = 7.7 Hz, 1H), 7.91 (d, *J* = 7.8 Hz, 1H), 7.80 (d, *J* = 6.8 Hz, 1H), 7.63 (s, 1H), 7.58 – 7.41 (m, 3H), 4.13 (s, 1H), 3.04 (d, *J* = 10.1 Hz, 1H), 2.76 (d, *J* = 10.1 Hz, 1H), 2.12 – 1.89 (m, 3H), 1.65 (d, *J* = 10.8 Hz, 1H), 1.52 (s, *J* = 15.4 Hz, 3H), 1.39 (d, *J* = 4.7 Hz, 3H). ES + MS: (M + H) 297.17. HPLC^*^ *t*_g_ = 4.57 min.

*(R)-N-*[(*1-methyl-2-oxo-1,2-dihydropyridin-4-yl*)*methyl*]*-1-*[*1-*(*naphthalen-1-yl*)*ethyl*]*piperidine-4-carboxamide* **(*13*).** The title compound was prepared as described for compound ***12*** from 4-(aminomethyl)-1-methylpyridin-2(1H)-one hydrochloride in 57% yield. ^1^H NMR (300 MHz, CDCl_3_) δ 8.42 (d, *J* = 7.1 Hz, 1H), 7.89 – 7.79 (m, 1H), 7.74 (d, *J* = 8.3 Hz, 1H), 7.57 (d, *J* = 5.7 Hz, 1H), 7.53 – 7.38 (m, 3H), 7.18 (d, *J* = 7.0 Hz, 1H), 6.36 (d, *J* = 1.1 Hz, 1H), 6.07 - 5.98 (m, 2H), 4.25 (d, *J* = 6.1 Hz, 2H), 4.17 - 4.05 (m, 1H), 3.48 (s, 3H), 3.23 (d, *J* = 12.6 Hz, 1H), 2.90 (d, *J* = 9.4 Hz, 1H), 2.27 – 1.66 (m, 7H), 1.46 (t, *J* = 7.5 Hz, 3H). ES + MS: (M + H) 404.2. HPLC^#^ *t*_g_ = 0.98 min.

*(R)-N-*[(*2-methylthiazol-5-yl*)*methyl*]*-1-*(*1-*(*naphthalen-1-yl*)*ethyl*)*piperidine-4-carboxamide* **(*14*).** The title compound was prepared as described for compound ***12*** from (2-methylthiazol-5-yl)methanamine in 25% yield. ^1^H NMR (400 MHz, DMSO) δ 8.44 (d, *J* = 8.0 Hz, 1H), 8.37 (t, *J* = 5.2 Hz, 1H), 7.91 (d, *J* = 7.3 Hz, 1H), 7.79 (d, *J* = 7.6 Hz, 1H), 7.58 – 7.42 (m, 4H), 7.39 (s, 1H), 4.33 (d, *J* = 5.7 Hz, 2H), 4.13 (q, *J* = 5.4 Hz, 1H), 3.04 (d, *J* = 8.8 Hz, 1H), 2.77 (d, *J* = 10.8 Hz, 1H), 2.56 (s, 3H), 2.14 – 1.91 (m, 3H), 1.66 (d, *J* = 12.0 Hz, 1H), 1.60 – 1.44 (m, 3H), 1.39 (d, *J* = 6.1 Hz, 3H). ES + MS: (M + H) 394.18. HPLC^*^ *t*_g_ = 5.20 min.

*(R)-1-*(*1-*(*naphthalen-1-yl*)*ethyl*)*-N-*(*pyridin-3-ylmethyl*)*piperidine-4-carboxamide* **(*15*).** The title compound was prepared as described for compound ***12*** from pyridin-3-ylmethanamine in 11% yield. ^1^H NMR (400 MHz, DMSO) δ 8.46 – 8.42 (m, 3H), 8.32 (t, *J* = 5.4 Hz, 1H), 7.91 (d, *J* = 7.4 Hz, 1H), 7.80 (d, *J* = 7.7 Hz, 1H), 7.62 – 7.42 (m, 5H), 7.32 (dd, *J* = 7.8, 4.8 Hz, 1H), 4.25 (d, *J* = 5.9 Hz, 2H), 4.15 (s, br, 1H), 3.06 (d, *J* = 10.1 Hz, 1H), 2.79 (d, *J* = 10.0 Hz, 1H), 2.21 – 2.08 (m, 1H), 2.01 (s, br, 2H), 1.71 (d, *J* = 13.1 Hz, 1H), 1.67 – 1.46 (m, 3H), 1.40 (d, *J* = 5.9 Hz, 3H). ES + MS: (M + H) 374.21. HPLC^*^ *t*_g_ = 4.93 min.

*(R)-1-*(*1-*(*naphthalen-1-yl*)*ethyl*)*-N-*(*4-sulfamoylbenzyl*)*piperidine-4-carboxamide formate* **(*16*).** The title compound was prepared as described for compound ***12*** from 4-(aminomethyl)benzenesulfonamide hydrochloride in 46% yield. ^1^H NMR (300 MHz, DMSO) δ 8.45 (d, *J* = 7.1 Hz, 1H), 8.35 (t, *J* = 5.7 Hz, 1H), 8.14 (s, 1H), 7.95 – 7.87 (m, 1H), 7.80 (d, *J* = 8.0 Hz, 1H), 7.74 (d, *J* = 8.4 Hz, 2H), 7.58 – 7.42 (m, 4H), 7.36 (d, *J* = 8.4 Hz, 2H), 7.28 (s, 2H), 4.28 (d, *J* = 5.9 Hz, 2H), 4.16 (q, *J* = 6.9 Hz, 1H), 3.08 (d, *J* = 10.6 Hz, 1H), 2.79 (d, *J* = 11.2 Hz, 1H), 2.23 – 1.94 (m, 3H), 1.73 (d, *J* = 13.5 Hz, 1H), 1.67 – 1.46 (m, 3H), 1.40 (d, *J* = 6.6 Hz, 3H). ES + MS: (M + H) 452.2 HPLC* *t*_g_ = 1.08 min.

*(R)-1-*(*1-*(*naphthalen-1-yl*)*ethyl*)*-N-*((*tetrahydro-2H-pyran-4-yl*)*methyl*)*piperidine-4-carboxamide* **(*17*).** The title compound was prepared as described for compound ***12*** from (tetrahydro-2H-pyran-4-yl)methanamine in 78% yield. ^1^H NMR (300 MHz, CDCl_3_) δ 8.42 (d, *J* = 7.5 Hz, 1H), 7.89 – 7.80 (m, 1H), 7.74 (d, *J* = 8.1 Hz, 1H), 7.57 (d, *J* = 7.1 Hz, 1H), 7.53 – 7.38 (m, 3H), 5.53 (s, 1H), 4.15 – 4.07 (m, 1H), 4.01 – 3.89 (m, 2H), 3.34 (td, *J* = 11.8, 2.2 Hz, 2H), 3.23 (d, *J* = 11.0 Hz, 1H), 3.14 (t, *J* = 6.4 Hz, 2H), 2.90 (d, *J* = 11.3 Hz, 1H), 2.18 – 1.94 (m, 3H), 1.94 – 1.62 (m, 6H), 1.62 – 1.51 (m, 2H), 1.47 (d, *J* = 6.6 Hz, 3H), 1.28 (qd, *J* = 12.1, 4.5 Hz, 2H). ES + MS: (M + H) 381.2. HPLC^#^ *t*_g_ = 1.07 min.

#### General Synthesis Compound 11

*(R)-1-(2-methoxypyridin-4-yl)-N-*((*1-*(*1-*(*naphthalen-1-yl*)*ethyl*)*piperidin-4-yl*)*methyl*)*methanamine formate* **(*11*)**. To a stirred solution of ***5c*** (200 mg, 0.50 mmol) in THF (10 ml) was added 2.5M lithium aluminium hydride in THF (595 μL, 1.49 mmol) at 0°C under N_2_. Then the reaction mixture was heated to 90°C and stirred for 5 h. After completion of the reaction (TLC Monitoring), the reaction mixture was quenched with 0.1 ml water, 0.1 ml 15% aq. NaOH and 0.2 ml water respectively at 0°C. The reaction mixture was filtered through Celite® and the filter cake washed with THF. The combined organics were dried over anhydrous Na_2_SO_4_ and concentrated in vacuo. The crude residue was purified by preparative HPLC to give the title compound in 14% yield. ^1^H NMR (400 MHz, DMSO) δ 8.43 (d, *J* = 7.2 Hz, 1H), 8.17 (s, 1H), 8.05 (d, *J* = 5.2 Hz, 1H), 7.94 – 7.87 (m, 1H), 7.79 (d, *J* = 8.0 Hz, 1H), 7.57 – 7.42 (m, 4H), 6.92 (d, *J* = 5.2 Hz, 1H), 6.75 (s, 1H), 4.15 (q, *J* = 6.5 Hz, 1H), 3.81 (s, 3H), 3.67 (s, 2H), 3.04 (d, *J* = 10.2 Hz, 1H), 2.74 (d, *J* = 11.1 Hz, 1H), 2.33 (d, *J* = 6.5 Hz, 2H), 2.05 – 1.93 (m, 2H), 1.72 (d, *J* = 12.6 Hz, 1H), 1.60 (d, *J* = 12.4 Hz, 1H), 1.45 – 1.33 (m, 4H), 1.15 – 0.99 (m, 2H). ES + MS: (M + H) 390.24. HPLC^^^ *t*_g_ = 4.78 min.

### Absorption, Distribution, Metabolism and Excretion Studies

#### Microsomal Stability and Metabolite Identification

The microsomal stability assay was performed by incubating compounds (1 µM) with human or mouse liver microsomes (0.4 mg/ml, Sekisui XenoTech, Kansas City, KS) suspended in 0.1 M phosphate buffer (pH 7.4) containing 1 U/mL glucose-6-phosphate dehydrogenase at 37°C. The metabolic reaction was initiated by the addition of an NADPH-regenerating system (final concentrations of 1.3 mM NADP, 3.5 mM glucose-6-phosphate, and 3.3 mM MgCl_2_). Control samples that did not include cofactor were also included. Samples were mixed and maintained at 37°C using a microplate incubator (THERMOstar®, BMG Labtech GmbH, Offenburg, Germany) and quenched at various time points over 60 min by the addition of acetonitrile containing metolazone as an internal standard. Quenched samples were centrifuged, and the supernatant removed and analyzed by LC/MS (Waters Xevo G2 QToF MS coupled to an Acquity UPLC) using a Supelco Ascentis Express RP C8 column (5 cm × 2.1 mm, 2.7 µm) and a mobile phase consisting of 0.05% formic acid in water and 0.05% formic acid in acetonitrile and mixed under gradient conditions. The flow rate was 0.4 ml/min and injection volume was 5 µL. The *in vitro* intrinsic clearance was calculated from the first order degradation rate constant for substrate depletion.

Metabolite identification was conducted with the assistance of Waters UNIFI software and candidate masses were filtered based on retention time, mass error and the response relative to that of the parent. The identity of an M+34 metabolite as the dihydrodiol on the naphthalene was confirmed by analysis of the CID spectrum. For other metabolites, identification was based on accurate mass only.

#### Kinetic Solubility

Kinetic solubility was determined based on a method described previously (Bevan and Lloyd, 2000). Test compounds prepared at 10 mg/ml in DMSO were diluted into buffer (pH 2.0 or pH 6.5) to give a 1% v/v final DMSO concentration. After standing for 30 min at ambient temperature, samples were analyzed via nephelometry to determine a solubility range. The maximum value of the assay is 100 μg/ml and the minimum value is 1.6 μg/ml.

#### Caco-2 Permeability

The apparent permeability coefficient was assessed using Caco-2 cell monolayers as described previously (Charman et al., 2020). Briefly, experiments were conducted over 120 min using an aqueous transport buffer (pH 7.4 Hanks balanced salt solution containing 20 mM HEPES) in both the apical and basolateral chambers. Propranolol (high permeability control), lucifer yellow (low permeability control) and rhodamine 123 (P-gp substrate) were used as controls. Donor solutions were prepared by spiking compound into transport buffer, equilibrating at 37°C for approximately 4 h, and centrifuging to remove any precipitated material. The supernatant was used as the donor solution and flux was assessed over 120 min. Samples were taken from the donor chamber at the start and end of the transport experiment, and from the acceptor chamber at 5-6 time points. The volume of acceptor solution removed was replaced with blank transport buffer and concentrations corrected for the dilution. Samples were stored frozen at -80°C until analysis by LC/MS as described below. The apparent permeability coefficient (P_app_) was calculated as P_app_ = (dQ/dt)/(Co x A), where dQ/dt is the rate of permeation across the cell monolayer, Co is the initial donor concentration and A is the monolayer surface area. P_app_ was measured in both the apical to basolateral (A-B) and basolateral to apical (B-A) directions and the efflux ratio was calculated as B-A P_app_/A-B P_app_. Mass balance was also confirmed.

#### Plasma Stability

Compound stability in mouse plasma was assessed in the absence and presence of 500 µM bis-*para*-nitrophenyl phosphate (BNPP), a known carboxylesterase inhibitor (Eng et al., 2010). Compound was spiked into blank mouse plasma (that had been pre-equilibrated with blank solvent or 500 µM BNPP at 37°C for 1 h) and maintained at 37°C under a humidified CO_2_-enriched (2%) atmosphere for pH control. Samples were collected at 0, 2, 4 and 6 h (*n* = 2 aliquots per time point) and snap frozen on dry ice and stored at -80°C until analysis by LC/MS.

#### Plasma Protein Binding

Mouse protein binding was determined via rapid equilibrium dialysis (RED) using a method modified from that reported previously (Curran et al., 2011). Mouse plasma (with 500 µM BNPP as a carboxylesterase inhibitor) was spiked with compound, mixed, and aliquots taken to determine the compound concentration in pre-dialysis matrix. The remaining spiked matrix was equilibrated at 37°C (∼10 min) prior to adding to the RED inserts (300 µL per insert). Inserts (*n* = 4 per compound) were placed in a Teflon holding plate and dialysed against protein-free 0.1 M phosphate buffered saline (pH 7.4; 500 µL per insert) at 37°C on an orbital plate shaker (ThermoMixer C, Eppendorf; 800 rpm). At the end of the 6 h dialysis period, aliquots were taken from the donor and dialysate chambers to obtain measures of the total and free concentrations, respectively. To control solution pH, the dialysis was performed in an incubator under a humidified CO_2_-enriched (2%) atmosphere and the pH of the post-dialysis matrix and dialysate were confirmed to be within pH 7.4 ± 0.1. The donor and dialysate samples were matrix matched (to a common composition of 50/50 plasma and buffer) and stored frozen at -80°C until analysis by LC/MS. The fraction unbound was determined as the ratio of the dialysate to donor concentration with the assumption that the system had reached steady state equilibrium at the end of the dialysis period.

#### LC/MS Analysis

Plasma protein binding and Caco-2 samples were assayed by LC/MS using a Waters Xevo TQ MS coupled to a Waters Acquity UPLC. The column was a Supelco Ascentis Express RP C8 column (5 cm × 2.1 mm, 2.7 µm) and the mobile phase consisted of 0.05% formic acid in water and 0.05% formic acid in acetonitrile mixed under gradient elution conditions with a 4 min cycle time, 0.4 ml/min flow rate and a 4 µL injection volume. Detection was conducted by electrospray ionization under positive and negative mode with multiple reaction monitoring. Diazepam was included as an internal standard and MS transitions included (m/z) 391.3 > 154.91 (3k), 404.23 > 250.18 (5c), 434.16 > 185.06 (9), and 285.04 > 193.07 (diazepam). The calibration standards were prepared in blank 50/50 plasma and buffer mixture (same matrix as the samples). Proteins were precipitated with acetonitrile (2:1 acetonitrile:matrix) and sample concentrations quantified by comparison to the calibration standards. Accuracy (% bias) and precision (%RSD) were within ±12% and <10%, respectively, for all compounds.

### Cell-Based Studies and Infection Assays

#### Cell Lines Used

Calu-3 and Vero (CCL-81) cells displayed expected cell morphologies and were sent for validation to Garvan Molecular Genetics facility (on 15 June 2020). Cell lines were screened on a monthly basis for *mycoplasma* contamination using the PlasmoTest kit (Invivogen) as per manufacturer’s instructions. All used cells were *mycoplasma* free.

#### Cell Culture

Calu-3 cells were cultured in Dulbecco’s Modified Eagle Medium F12 supplemented with 10% (v/v) heat-inactivated foetal bovine serum (FBS; Sigma-Aldrich), 100 U/ml penicillin and 100 mg/ml streptomycin at 37°C and 5% CO_2_.

For infection studies, Vero (CCL-81) cells were cultured in Dulbecco’s Modified Eagle Medium (DMEM + 1 g/L D-Glucose, L-Glutamine and 110 mg/L Sodium Pyruvate; Gibco) supplemented with 10% (v/v) heat-inactivated fetal bovine serum (FBS; Sigma-Aldrich), 100 U/mL penicillin and 100 mg/ml streptomycin at 37°C and 5% CO_2_.

#### SARS-CoV-2 Infection and Inhibitor Treatment

SARS-CoV-2 was obtained from The Peter Doherty Institute for Infection and Immunity (Melbourne, Australia), where the virus was isolated from a traveller from Wuhan arriving in Melbourne and admitted to hospital in early 2020. Viral material was used to inoculate Vero/hSLAM cells for culture, characterisation and rapid sharing of the isolate (Caly et al., 2020).

For infection assays Calu-3 cells were seeded in a volume of 100 μL DMEM F12 into tissue culture-treated flat-bottom 96-well plates (Falcon) at a density of 3.5 × 10^4^ cells/well and incubated over night before infection and/or treatment at confluency. On day of infection and/or treatment cells were washed twice with serum free DMEM medium and infected with SARS-COV-2 and MOI of 0.1 in 25 μL of serum-free medium containing TPCK trypsin (0.5 μg/ml working concentration, ThermoFisher). Cells were cultured at 37°C and 5% CO_2_ for 30 min. Cells were topped up with 150 µL of medium containing PLpro inhibitor compounds at indicated concentrations in 6 replicates per concentration. At 48 h post infection/treatment, 100 μL of supernatant was harvested from each well and kept frozen at -80°C.

#### Median Tissue Culture Infectious Dose (TCID50) Assay

For TCID50 assays, Vero cells were seeded in a volume of 100 µL DMEM medium into tissue culture treated flat-bottom 96-well plates (Falcon) at a density of 1 × 10^4^ cells/well and incubated overnight. The next day, Vero plates were washed twice with PBS and 125 µL of DMEM +100 U/mL penicillin and 100 mg/ml streptomycin (serum free) + TPCK trypsin (0.5ug/ml working conc) was added and kept at 37°C, 5% CO_2_. Calu-3 cell supernatants were thawed and serial 1:7 dilutions prepared in 96-well round bottom plates at 6 replicates per dilution. 25 µL of serially diluted calu-3 supernatant were added onto Vero cells and plates incubated for 4 days at 37°C, 5% CO_2_ before measuring cytopathic effect under a light microscope. The TCID50 calculation was performed using the Spearman and Kärber method.

## Data Availability

The datasets presented in this study can be found in online repositories. The names of the repository/repositories and accession number(s) can be found below: Protein Data Bank, ID: 7TZJ.

## Supplementary Material

Supplementary Figure S1
**(A)** The screening data quality for the ReFRAME library screen was monitored by the robust Z’, calculated on 128 negative and 128 positive control wells for each assay plate. All plates had a robust Z’ > 0.5 with an average value of 0.65 across 9 plates. **(B)** Signal to background (S/B) ratio for each of the plates screen. S/B was consistent with an average of 3 across all plates screened. **(C)** Summary of screen data expressed at % inhibition. The hit selection criteria was defined as compounds where % inhibition was >2.5* MAD above the average of the negative control. There were 789 compounds identified using these criteria. 53 compounds were selected and the potency was determined in 10-point titrations. **(D)** 10-point titration for 53 compounds identified from a HTS of the ReFRAME library. Compounds were assayed at a top concentration of 100 µM and titrated using 1:2 (Plpro) or 1:3 (USP21) serial dilutions. 100 µM ***rac5c*** was used as a positive control for the HTS. The top 12 most potent compounds are also shown in Figure 1B.

Supplementary Figure S2 (A) Dose response curves for ***rac5c, 5c, rac3k*** and ***3k.*** The (R)-enantiomers of both ***5c*** and ***3k*** were synthesized and their activity was compared against their racemic counterparts. The inhibitory activity was tested in two independent experiments (*n* = 2) each containing two technical replicates using the HTS assay. Individual data points represent the mean replicate value for each experiment. **(B)** Chemical structure and dose response curve for the literature compound ***GRL-0617.*** The inhibitory activity was tested in two independent experiments (*n* = 2) each containing two technical replicates using the HTS assay. We observed a similar IC_50_ to reports in other studies (Fu et al., 2021; Ma et al., 2021; Osipiuk et al., 2021; Shen et al., 2021). This confirms observations seen with SARS-CoV that ***5c*** remains a more potent inhibitor PLpro. Individual data points represent the mean replicate value for each experiment.

Supplementary Figure S3
**(A)** Electron density map (2|Fo| – |Fc|) at 1 σ for the asymmetric unit. SARS-CoV-2 PLpro is shown as a ribbon and ***3k*** is shown in stick representation. **(B, C)** Electron density map (2|Fo| – |Fc|) at 1 σ of the ligand binding site for ***3k*** in molecule A (chain A) or for molecule B (chain B), with key interacting/changed residues indicated. In both chains, clear electron density can also be seen atop the ligand that was modelled as a single molecule of DMSO. The DMSO is held in place by interacting with the carbonyl in ***3k***.

Supplementary Figure S4 To ensure the SARS-CoV-2 PLpro BL (PLpro^BL^) mutant was active prior to calculating dose response curves, triubiquitin (top) and pro-ISG15 (bottom) cleavage was followed over time and visualised on Coomassie-stained SDS-PAGE gels [see Methods and (Klemm et al., 2020)]. PLpro^BL^ retained activity, though was less active compared to wild-type PLpro. A catalytically dead PLpro (PLpro^C111S^) was used as a negative control for the assay.

Supplementary Figure S5 (A) *3k* was tested for specificity to inhibit SARS-CoV PLpro, MERS-CoV PLpro, SARS-CoV-2 PLpro, or SARS-CoV-2 PLpro with residues 267-272 of blocking loop 2 (BL2) substituted for those in MERS-CoV (SARS-CoV-2^BL^) in a UbRh assay. Inhibitor ***3k*** is cross-reactive with SARS-CoV PLpro and SARS-CoV-2 PLpro but not with MERS-CoV PLpro. These data indicate that engaging the conserved BL2 is crucial for the inhibition of PLpro by ***3k***. Experiments were performed using the HTS assay as two independent experiments (*n* = 2) each containing two technical replicates. Individual data points represent the mean replicate value for each experiment. **(B)** SPR assays for compound ***3k*** against PLpro variants from Figure 3B. The top panels show double referenced sensorgram data as a function of time and the bottom show steady-state dose response curves. As for ***5c***, absence of inhibitor reactivity with MERS-CoV PLpro can be explained by a loss in direct binding. Binding was measured by detecting changes in response units (RU) of the sensor chip. All SPR experiments were performed in triplicate; a representative example is shown. See Supplementary Table S1 for all data and Supplementary Figure S9 for the response curves of the remaining experiments.

Supplementary Figure S6 Dose response curves used to calculate the inhibitory activity of the selected compounds shown in Figure 5. Experiments were performed using the HTS assay as two independent experiments (*n* = 2) each containing two technical replicates. Individual data points represent the mean replicate value for each experiment.

Supplementary Figure S7 (A)Chemical structure and dose response curve for the literature compound ***18*** identified as compound ***19*** in the corresponding publication (Shan et al., 2021). The inhibitory activity was tested in four independent experiments (*n* = 4) each containing two technical replicates using the HTS assay and does not appear to improve on that seen for ***5c*** in our hands. Individual data points represent the mean replicate value for each experiment. **(B)** SPR assay for literature compound ***18*** against SARS-CoV-2 PLpro. The top panels show double referenced sensorgram data as a function of time and the bottom show steady-state dose response curves. No direct binding assays were performed in (Shan et al., 2021) and here we show the affinity for compound ***18*** lies within the range of ***5c*** and does not appear to improve on the binding affinity with PLpro. Binding was measured by detecting changes in response units (RU) of the sensor chip. All SPR experiments were performed in triplicate; a representative example is shown. See Supplementary Table S1 for all data and Supplementary Figure S9 for the response curves of the remaining experiments.

Supplementary Figure S8 (A) *9* was tested for specificity to inhibit SARS-CoV PLpro, MERS-CoV PLpro, SARS-CoV-2 PLpro, or SARS-CoV-2 PLpro with residues 267-272 of blocking loop 2 (BL2) substituted for those in MERS-CoV (SARS-CoV-2^BL^) in a UbRh assay. Inhibitor ***9*** is cross-reactive with SARS-CoV PLpro and SARS-CoV-2 PLpro but not with MERS-CoV PLpro. The presence of a methoxy on the naphthalene appears to favour improved metabolic stability rather than affecting direct binding with PLpro. Experiments were performed using the HTS assay as four independent experiments (*n* = 4) each containing two technical replicates. Individual data points represent the mean replicate value for each experiment. **(B)** SPR assays for compound ***9*** against PLpro variants from Figure 3B **.** The top panels show double referenced sensorgram data as a function of time and the bottom show steady-state dose response curves. The presence of a methoxy on the naphthalene appears to favour improved metabolic stability rather than affecting direct binding with PLpro. Binding was measured at steady-state by detecting changes in response units (RU) of the sensor chip averaging over 5 s prior to the end of the analyte injection. All SPR experiments were performed in triplicate; a representative example is shown. See Supplementary Table S1 for all data and Supplementary Figure S9 for the response curves of the remaining experiments.

Supplementary Figure S9 All compounds tested by SPR were performed in triplicate as independent experiments. This Panel shows the remaining two dose response curves for the SPR assays noted throughout the main text and in Supplementary Table S1. The mean value of all three experiments ± SD is reported throughout the text.

Supplementary Table S1 SPR affinities determined at steady state for three independent experiments (Exp1, Exp2 and Exp3) reporting mean and 1 SD for each experiment group. Dose response curves are shown in Figure 3C, Supplementary Figures S5, S7–S9 **.**
