# Supplementary material for: Insights Into Drug Repurposing, as Well as Specificity and Compound Properties of Piperidine-Based SARS-CoV-2 PLpro Inhibitors
Source: Front Chem. 2022 Apr 12;10:861209. doi: 10.3389/fchem.2022.861209 (PMC9039177; doi:10.3389/fchem.2022.861209)
Supplement: Supplementary file 1 [file Table1.pdf]

Supplementary Table S1. SPR affinities determined at steady state for three independent experiments (Exp1, Exp2 and Exp3) reporting mean and 1 standard deviation for each experiment group. Dose response curves are shown in **Figures 3C**, **S5, S7-S9.**

		K_D (μM)				
**PLpro**	**Compound**	*Exp1*	*Exp2*	*Exp3*	**Mean**	**SD**
SARS-CoV-2 $PLpro^{WT}$	***3k***	*2.00*	*2.35*	*2.85*	**2.40**	0.43
SARS-CoV-2 $PLpro^{WT}$	***5c***	*4.11*	*2.45*	*2.60*	**3.05**	0.92
SARS-CoV-2 $PLpro^{WT}$	***9***	*1.42*	*1.82*	*2.34*	**1.86**	0.46
SARS-CoV-2 $PLpro^{WT}$	***18***	*1.14*	*1.40*	*1.64*	**1.39**	0.25
SARS-CoV	***3k***	*0.662*	*0.795*	*0.686*	**0.714**	0.071
SARS-CoV	***5c***	*0.833*	*0.880*	*0.853*	**0.855**	0.024
SARS-CoV	***9***	*0.805*	*0.800*	*0.840*	**0.815**	0.022
SARS-CoV	***18***	*0.311*	*0.320*	*0.320*	**0.317**	0.005
MERS-CoV	***3k***	*>20*	*>20*	*>20*	**ND**	
MERS-CoV	***5c***	*>20*	*>20*	*>20*	**ND**	
MERS-CoV	***9***	*>20*	*>20*	*>20*	**ND**	
MERS-CoV	***18***	*>20*	*>20*	*>20*	**ND**	
SARS-CoV-2 $PLpro^{BL}$	***3k***	*>20*	*>20*	*>20*	**ND**	
SARS-CoV-2 $PLpro^{BL}$	***5c***	*>20*	*>20*	*>20*	**ND**	
SARS-CoV-2 $PLpro^{BL}$	***9***	*>20*	*>20*	*>20*	**ND**	
SARS-CoV-2 $PLpro^{BL}$	***18***	*>20*	*>20*	*>20*	**ND**	
